# GATA6 enhances the stemness of human colon cancer cells by creating a metabolic symbiosis through upregulating *LRH‐1* expression

**DOI:** 10.1002/1878-0261.12647

**Published:** 2020-02-26

**Authors:** Hung‐Tzu Lai, Chin‐Ting Chiang, Wen‐Ko Tseng, Ta‐Chung Chao, Yeu Su

**Affiliations:** ^1^ Institute of Biopharmaceutical Sciences School of Pharmaceutical Sciences National Yang‐Ming University Taipei Taiwan, R.O.C.; ^2^ Program in Molecular Medicine School of Life Sciences National Yang‐Ming University Taipei Taiwan, R.O.C.; ^3^ Colorectal Surgery Department Chung‐Gung Memorial Hospital, Keelung Branch Taiwan, R.O.C.; ^4^ Division of Medical Oncology Department of Oncology Taipei Veterans General Hospital Taiwan, R.O.C.; ^5^ Faculty of Medicine School of Medicine National Yang‐Ming University Taipei Taiwan, R.O.C.

**Keywords:** colon cancer, GATA6, LRH‐1, metabolic symbiosis, stemness

## Abstract

Cancer stem cells play critical roles in tumor initiation, progression, and relapse. Since we previously found that GATA6 promotes the stemness in HCT‐116 and HT‐29 human colorectal cancer (CRC) cells, we aimed to identify the downstream mediator(s) of the stemness‐stimulating effect of GATA6 herein. LRH‐1 was found as a direct target of GATA6 and its upregulation promoted the stemness in both HCT‐116 and HT‐29 cells. Subsequently, hypoxia‐inducible factor‐1α (HIF‐1α) was identified as a direct target of LRH‐1 and its expression level and activity were significantly elevated in the LRH‐1‐overexpressing clones established from the aforementioned two CRC lines. Accordingly, the expression levels of several HIF‐1α targets were also markedly increased, resulting in a stronger glycolysis associated with dramatic elevations of the lactate levels in these cells. Strikingly, higher mitochondrial activities were also found in these clones which might be attributed to the increase of PGC‐1α stimulated by the lactate uptaken through the upregulated MCT‐1. Finally, significant increases in the self‐renewal ability, intracellular radical oxygen species levels and mitochondrial mass were detected in the CD133^+^/CD44^+^ subpopulations isolated from CRC cells regardless of their LRH‐1 expression levels. Together, our results suggest a novel metabolic symbiosis between different colorectal cancer stem cell subpopulations critical for maintaining their mutual stemness.

## Graphical abstract

Our observations demonstrate a reverse Warburg effect in human colorectal cancer stem cells (CRCSCs) because the expression levels and activity of hypoxia‐inducible factor‐1α are markedly increased by LRH‐1 upregulation resulted from GATA6 overexpression in the majority of colorectal cancer (CRC) cells which undergo aerobic glycolysis to proliferate rapidly and, in the meantime, generate large amount of lactate that can be uptaken by another subset of CRC cells for mitochondrial oxidative phosphorylation. In this manner, metabolic symbiosis and energy transfer are maintained between two CRCSC subpopulations.

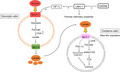

**Abbreviations**
ALDH: aldehyde dehydrogenase
CRCSCs: colorectal cancer stem cells
ECAR: extracellular acidification rate
GATA6: GATA binding protein 6
HIF‐1α: hypoxia‐inducible factor 1‐alpha
LRH‐1: liver receptor homolog‐1
MCT‐1: Monocarboxylate transporter 1
NAO: nonyl acridine orange
OCR: oxygen consumption rate
OXPHOS: oxidative phosphorylation
PGC‐1α: peroxisome proliferator‐activated receptor gamma coactivator 1‐alpha

## 1 Introduction

Colorectal carcinoma (CRC) is the third and second most common type of cancer in male and female, respectively, as 1.8 million new cases and 862 000 deaths worldwide are estimated in 2018 (Bray *et al.*, 2018). Although a variety of options for CRC treatment such as traditional chemotherapeutic drugs, 5‐FU, oxaliplatin, and irinotecan (Carethers, 2008), monoclonal antibodies against vascular endothelial growth factor and epidermal growth factor receptor, as well as a multikinase inhibitor (i.e., regorafenib) (Loree and Kopetz, 2017), are available, the 5‐year survival rate of the patients with metastatic CRC (mCRC) remains low (Zeki *et al.*, 2011).

Accumulating evidence supports the idea that cancer metastasis and recurrence are caused by a small subset of cancer cells with stem cell‐like features, termed cancer stem cells (CSCs) (Dick, 2003; Wang and Dick, 2005). The presence of colorectal cancer stem cells (CRCSCs) in patients' tumor tissues was first identified by the use of CD133 as a surface marker (O'Brien *et al.*, 2006; Ricci‐Vitiani *et al.*, 2006). Later on, CD44 (Dalerba *et al.*, 2007), Lgr5 (Barker *et al.*, 2008), and others (Botchkina, 2013) were also found to be the markers for CRCSCs. In addition, some aldehyde dehydrogenase (ALDH)‐overexpressing tumor cells were considered to be CRCSCs because this enzyme could transduce crucial signals to enhance the stemness of these cells (Tomita *et al.*, 2016). Since successful eradication of CSCs was considered to be necessary for curing cancer, a better understanding of the mechanisms through which CSCs drive CRC progression may provide new treatment strategy.

In our previous work, we found that GATA6 upregulation could enhance the stemness properties in HCT‐116 and HT‐29 human CRC cells (Lai *et al.*, 2020). GATA6 is a member of the GATA transcription factor family that plays critical roles in embryonic development (Molkentin, 2000). Moreover, different members of this family form a network to maintain the embryonic stem cells in an undifferentiated state through regulating LIF and Oct3/4 (Zhang *et al.*, 2007). Interestingly, GATA6 appears to be required for crypt cell proliferation, secretory cell differentiation, and absorptive enterocyte gene expression in the small intestine (Beuling *et al.*, 2011). Additionally, overexpression of GATA6 has been detected in colorectal polyps as well as primary and metastatic tumors which might promote the invasion of human CRC cells by activating the promoter of urokinase‐type plasminogen activator (uPA) (Belaguli *et al.*, 2010). To further identify the downstream mediator of the stemness‐promoting effects of GATA6, we knocked down the expression of either *LRH‐1* (liver receptor homolog‐1) or *Hes‐1* (hairy and enhancer of split‐1) in the GATA6‐overexpressing clones because both were potential targets of GATA6 (Sulahian *et al.*, 2013) and found that silencing the expression of either one is sufficient to diminish the self‐renewal abilities of these clones (Lai *et al.*, 2020). Since the involvement of Hes‐1 in stimulating the stemness of human CRC cells has already been demonstrated (Gao *et al.*, 2014), we herein focused on dissecting the stemness‐enhancing role of LRH‐1 in human CRC cells.

LRH‐1 (also known as NR5A2) belongs to the nuclear receptor (NR) family which contains many transcription factors that regulate diverse biological processes such as embryonic development, differentiation, metabolism, and cancer (Simandi *et al.*, 2013). Accumulating evidence indicates that LRH‐1 participates in the pathogenesis of multiple tumors including pancreatic (Benod *et al.*, 2011; Petersen *et al.*, 2010), breast (Thiruchelvam *et al.*, 2011), gastric (Wang *et al.*, 2008), and colon cancer (Schoonjans *et al.*, 2005). For example, LRH1 has been shown to enhance breast cancer cell chemoresistance by upregulating DNA damage checkpoint 1 (MDC1) and attenuating DNA damage (Wang *et al.*, 2018). In addition, LRH‐1 has recently been reported to suppress p53 action at the *p21* gene, allowing CRC cells to evade p21‐mediated cell cycle arrest (Kramer *et al.*, 2015). More interestingly, a prominent role of LRH‐1 in maintaining stem cell properties and in the regulation of EMT in pancreatic CSCs has been demonstrated (Luo *et al.*, 2017).

In this study, we found that LRH‐1 is a direct target of GATA6, and its upregulation can promote the stemness properties in HCT‐116 and HT‐29 human CRC cells. Furthermore, we demonstrated that LRH‐1 overexpression in these cells results in the emergence of two CRCSC subpopulations with a metabolic symbiosis between them.

## 2 Materials and methods

### 2.1 Cell culture

Human colorectal carcinoma (CRC) cell lines HCT‐116 and HT‐29 were purchased from the American Type Culture Collection (ATCC, Manassas, VA, USA), and their identities were validated by STR profiling (Bioresource Collection and Research Center, Taiwan). The two CRC lines were maintained in RPMI‐1640 (Life Technologies, Carlsbad, CA, USA) supplemented with 10% FBS (Gibco, Waltham, MA USA), 100 units·mL^−1^ penicillin, 100 µg·mL^−1^ streptomycin, and 25 µg·mL^−1^ amphotericin B (PSA, Biological Industries, Kibbutz Beit Haemek, Israel) at 37 °C and 5% CO_2_ and were routinely tested for mycoplasma. Several stable clones established, respectively, from HCT‐116 (116 Vec, OED, and OEJ) and HT‐29 (29 Vec, OE7, and OE8) cells were maintained under the aforementioned conditions except that proper concentrations of puromycin (Invitrogen, Carlsbad, CA, USA) were added.

### 2.2 Plasmid constructions

To generate a reporter capable of responding to GATA6, two primers (sense: 5′‐AAAAGGTACCACATATTCACCATTT‐3′ and antisense: 5′‐AAAACTCGAGGCC TTGGGAAGGACA‐3′) were used to amplify the promoter region (−599 to +170) of *LRH‐1* by PCR using the genomic DNA isolated from HCT‐116 cells as a template. PCR‐mediated mutagenesis was then performed to generate a *LRH‐1* promoter with mutations in the two furthest putative GATA6‐binding sites using the following primer set (forward: 5′‐AAAGGTACCGGGTATAAAGATATAGAT‐3′ and reverse: 5′‐AA AACTCGAGGCCTTGGGAAGGACA‐3′). The aforementioned PCR fragments were then inserted into the KpnI and XhoI sites of the pGL3‐basic vector (Promega, Madison, WI, USA) to generate pLRH‐1w‐luc and pLRH‐1m‐luc, respectively. To generate the LRH‐1 expression plasmid, its cDNA fragment was amplified by PCR using pTetOLRH‐1 (a kind gift from C. D. Clyne of Cancer Drug Discovery Laboratory, Prince Henry's Institute of Medical Research, Clayton, Victoria 3168, Australia) as a template with primer set (forward: 5′‐AAAAGAATTCATGCTGCCC AAAGTG‐3′ and reverse: 5′‐AAAAGTCGACTTATGCTCTTTTGGC‐3′) and then subcloned into the KpnI and SaII sites of the pBabe vector (National RNAi Core of Academia Sinica, Taiwan). To generate a reporter able to respond to LRH‐1, two primers (sense: 5′‐AAAGGTACCACTCTTTCCCTGAAAT‐3′ and antisense: 5′‐AA ACTCGAGCTGAGAAGGGATTTCG‐3′) were used to amplify the promoter region of *HIF‐1α* (−1204 to −687) which contains a putative LRH‐1‐binding motif by PCR using the genomic DNA isolated from HCT‐116 cells as a template. PCR‐mediated mutagenesis was then performed to generate the aforementioned DNA fragment except containing a mutated LRH‐1‐binding motif using following primer set (forward: 5′‐ACTTAGTAGACATCGTGAGTTCCCCT‐3′ and reverse: 5′‐AGGGGAACTCACG ATGTCTACTAAGT‐3′). These PCR fragments were then subcloned into the KpnI and XhoI sites of the pGL3‐control vector (Promega) to generate pHIF‐1αwt‐luc and pHIF‐1αmut‐luc, respectively. To generate a reporter responding to HIF‐1α, a DNA fragment containing five repeats of hypoxia response element (5xHRE) was cut out from the 5HRE/GFP (a kind gift from J.‐H. Liu of National Yang‐Ming University, Taiwan) by Hind III and XhoI and then subcloned into the pGL3‐basic vector to obtain p5xHRE‐luc.

### 2.3 Reporter assay

For reporter assay, 293T cells (5 × 10^4^ per well) seeded onto 24‐well plates were cotransfected with different amounts of (1.5 and 3 µg) GATA6‐expressing plasmid (OriGene Technologies, Rockville, MD, USA), 1 µg pLRH‐1w‐luc or pLRH‐1m‐luc plus 1 µg pCMV‐Laz (Clontech Laboratories, Mountain View, CA, USA, as an internal control for normalizing the transfection efficiency) as follows. Briefly, a DNA mixture was added into serum‐free DMEM to make a final volume of 100 µL. Meanwhile, 7.5 µL of PolyJet™ reagent (Signagen Laboratories, Rockville, MD, USA) was mixed with serum‐free DMEM for making a final volume of 100 µL. The mixture containing the aforementioned two reagents was placed at room temperature for 15 min before being added into the cells. After 16‐h incubation, the culture medium was replaced with a fresh DMEM containing 10% FBS and 1% PSA. Total lysates, prepared 48 h post‐transfection, were used to examine the activities of luciferase and β‐galactosidase, respectively. To examine the former, each lysate was diluted into 40µL lysis buffer (Promega) and reacted with 100 µL luciferase reagent buffer (Promega). Bioluminescence was then measured with a Mutilabel Counter (Wallac 1420 VICTPR^2^™; PerkinElmer, Waltham, MA, USA). The activity of β‐galactosidase was analyzed using 10 µg lysate in the presence of 4 mg·mL^−1^ *o*‐nitro‐phenyl‐β‐d‐galacto‐pyranoside (ONPG; Sigma‐Aldrich, St. Louis, MO, USA) for 30 min before OD420 being measured with a spectrophotometer (Bio‐Rad, Hercules, CA, USA). Similar protocols were applied to analyze the luciferase activities after the 116 Vec and OED clones were transfected, respectively, with pHIF‐1αwt‐luc or p5xHRE‐luc and pHIF‐1αwt‐luc, pHIF‐1αmut‐luc, or p5xHRE‐luc.

### 2.4 Chromatin immunoprecipitation

Chromatin immunoprecipitation (ChIP) assay was performed following the protocol described previously (Carey *et al.*, 2009). Briefly, cells (5 × 10^7^) were seeded in 100‐mm dishes overnight before being fixed with 1% formaldehyde for 10 min, followed by a 5‐min treatment with 1.25 m glycine to quench the reaction. The cell pellet was then resuspended in a cell lysis buffer [5 mm PIPES, pH 8.0; 85 mm KCl; 0.5% nonidet P‐40 (NP‐40)] and incubated on ice for 10 min before the nuclei were collected by centrifugation at 157 ***g*** for 10 min at 4 °C. Subsequently, the nuclei were resuspended in 1 mL of nuclei lysis buffer (50 mm Tris/Cl, pH 8.0; 10 mm EDTA; 1% SDS) supplemented with a protease inhibitor cocktail (Roche Molecular Biochemicals, Mannheim, Germany) and then incubated on ice for 10 min. Sonication was then applied to shear DNA into 500‐bp fragments, and precleared lysates were subjected to overnight immunoprecipitation with 2 µg·mL^−1^ of rabbit anti‐GATA6 antibody or normal rabbit IgG (negative control). After phenol/chloroform extraction, DNA samples were recovered by isopropanol precipitation and aliquots from the resuspended DNA pellets were subjected to a SYBR green‐based quantitative PCR assay using primers (Table S1) with a MiniOpticonTM Real‐Time PCR Detection System (Bio‐Rad).

### 2.5 Lentivirus preparation

Recombinant virus carrying the LRH‐1 cDNA was produced from 293T cells by transfected simultaneously with three plasmids. Briefly, 1 day before transfection, 8 × 10^5^ cells were seeded onto 60‐mm dishes, a DNA mixture containing 0.25 µg pMDG, 2.5µg pCLampho, and 2.25 µg LRH‐1‐expressing plasmids was mixed with the PolyJet™ reagent before being added into the cells as above described. After 16‐h incubation, the culture medium was replaced with a serum‐free medium containing 1% BSA. Viruses present in the media were collected by centrifugation at 24 and 48 h postmedia change, respectively, and then combined before being stored in aliquots at −70 °C.

### 2.6 Generation of the stable clones

The LRH‐1‐overexpressing stable clones from HCT‐116 (OED and OEJ) and HT‐29 (OE7 and OE8) cells were established by infecting them with the aforementioned lentiviruses. Single clones were isolated and expanded after the infected HCT‐116 and HT‐29 cells were cultured in selection media containing 1.5 and 1 µg·mL^−1^ of puromycin, respectively.

### 2.7 Real‐time RT‐PCR

Total RNAs extracted from the different clones derived from HCT‐116 and HT‐29 cells were reverse‐transcribed using MMLV reverse transcriptase (Thermo Fisher Scientific, Waltham, MA USA). SYBR green‐mediated PCR amplification assays were then conducted using the cDNAs as described above. The primer sets were designed to analyze the mRNA levels of specific genes (Table S1) and the reaction conditions were as follows: 95 °C for 30 s, 65 °C for 30 s, and 72 °C for 30 s. The relative expression levels of the target genes were calculated using the comparative Ct method (△CT) which were normalized against endogenous levels of GAPDH. Data processing was performed using cfx manger, version 2.1 (Bio‐Rad).

### 2.8 Western blotting

For preparing total lysates, cells were first washed with and then scraped into cold PBS supplemented with a proteinase inhibitor cocktail. After centrifugation, cell pellets were lysed in RIPA buffer (50 mm Tris/HCl, 150 mm NaCl, 0.1% SDS, 1% NP‐40, and proteinase inhibitor; pH 7.4). Total lysates (30 µg protein/well) were separated on a 10% SDS/polyacrylamide gel and then processed for immunoblotting. The primary antibodies including mouse anti‐LRH‐1 (Abcam, Cambridge, UK; ab41901), rabbit anti‐LGR5 (Abcam; ab75850), mouse anti‐HIF‐1α (Abcam; ab1), rabbit anti‐ALDH‐1 (Genetex, Irvine, CA, USA; GTX123973), mouse anti‐OXPHOS cocktail (Abcam; ab110413), rabbit anti‐PGC‐1α (Santa Cruz, Dallas, CA, USA; sc‐13067), mouse anti‐β‐actin (Merck Millipore, Burlington, MA, USA; MAB1501), rabbit anti‐GAPDH (Genetex; GTX100118) were properly diluted and then incubated with the blots at 4 °C overnight, followed by incubation with the horseradish peroxidase‐conjugated secondary antibodies.

Finally, signals were detected using an enhanced chemiluminescence system (ECL, NEN Life Science, Boston, MA, USA) and their intensities were quantified by densitometry (imagej, Betheada, MD, USA).

### 2.9 Sphere‐forming assay

Sphere‐forming assay was conducted by seeding 5 × 10^4^ cells into Petri dishes containing serum‐free RPMI supplemented with N2 (Thermo Fisher Scientific), 20 ng·mL^−1^ epidermal growth factor (R&D System, Minneapolis, MN, USA), 10 ng·mL^−1^ basic fibroblast growth factor (R&D System), 6 mg·mL^−1^ glucose, and 4 mg·mL^−1^ BSA. Two weeks later, spheres stained with MTT (1 mg·mL^−1^) were counted using metamorph software (Molecular Devices, San Jose, CA, USA).

### 2.10 Soft agar colony‐forming assay

Briefly, cells (5 × 10^4^) were resuspended in 2.7 mL RPMI medium and then mixed with 0.3 mL of prewarmed 3% agarose (dissolved in medium) and then were layered into three different wells of a 6‐well plate precoated with 1 mL of 0.6% agarose (dissolved in medium). After a 3‐week incubation, during which the media were replenished every 3 days, the colonies were stained with MTT (1 mg·mL^−1^) and then counted using metamorph software.

### 2.11 Detection of the CD133^+^/CD44^+^ subpopulations

Cells (1 × 10^6^) detached from the dishes by TrypLE™ Select Enzyme (Thermo Fisher Scientific) were incubated simultaneously with both the PE‐labeled anti‐CD133 antibody (Miltenyi Biotec, Bergisch Gladbach, Germany; 293C3) and the FITC‐labeled anti‐CD44 antibody (Beckman Coulter, Brea, CA, USA; IM1219U) in complete medium for 60 min on ice. After centrifugation, cells were washed several times with PBS and then resuspended in 1 mL PBS. Flow cytometry (BD FACSCanto™; Becton Dickinson, Franklin Lakes, NJ, USA) was used to analyze the percentage of CD133^+^/CD44^+^ subpopulations in different clones.

### 2.12 Intracellular hypoxia assay

Intracellular hypoxia was measured using the ROS‐ID^®^ Hypoxia/Oxidative stress detection kit (Enzo Life Sciences, Farmingdale, New York, USA). Cells were cultured in a 12‐mm glass‐bottom dish (4 × 10^5^ cells/dish) for 48 h before being treated with the kit reagent mix according to the manufacturer's instruction. Thirty min later, cells were washed twice with PBS before the fluorescent images were visualized by a fluorescence microscope (OlympusBX50; OLYMPUS, Tokyo, Japan). In addition, the hypoxic and oxidative subpopulations were detected by flow cytometry using OD580/595 nm and OD504/524 nm, respectively.

### 2.13 Determination of the lactate levels

Lactate levels were analyzed using the l‐lactate colorimetric assay kit (Abcam; ab65331) according to the manufacturer's instruction. Briefly, culture media as well as cell lysates were harvested from various clones 48 h postseeding and then mixed with the lactate assay buffer, substrate mix and enzyme mix provided by the kit. Subsequently, the OD450 of each sample was measured by a spectrophotometer (Multiskan Spectrum; Thermo Fisher Scientific) and the amount of lactate was calculated according to a standard curve and then further normalized with the cell number.

### 2.14 Measurement of the intracellular radical oxygen species

The intracellular radical oxygen species (ROS) levels were measured primarily as described (Wang and Joseph, 1999). In brief, cells (5 × 10^5^) were seeded in 60‐mm dishes for 24 h before being harvested and then incubated with 1 mm DCFH‐DA (Sigma‐Aldrich) at 37 °C for 30 min in the dark. The fluorescence of the oxidized products of DCFH‐DA was analyzed by flow cytometry using OD480/530 nm.

### 2.15 Seahorse metabolic analysis

Cells harvested from various clones were seeded on Seahorse XFe24 cell culture microplates (Agilent, Santa Clara, CA, USA; 100777‐004) for 24 h. Growth media were then changed to Seahorse XF Base Medium (Agilent; 102353‐100) supplemented with 200 mm l‐glutamine before being placed in a non‐CO_2_ incubator for 1 h. Extracellular acidification rate (ECAR) and oxygen consumption rate (OCR) were measured, respectively, using the glycolytic stress test assay (Agilent; 103030‐100) and mitochondrial stress test assay (Agilent; 103015‐100) in a Seahorse XFe24 Analyzer (Agilent) following the manufacturer's protocols. The glycolytic rate and mitochondrial respiration rate were then calculated by Wave software (Agilent) and normalized with the cell number.

### 2.16 Measurement of mitochondrial mass

Mitochondrial mass was quantified by flow cytometry after cells were stained with 100 nm nonyl acridine orange (NAO; Thermo Fisher Scientific) at 37 °C for 15 min, and data were subsequently analyzed using a flowjo software (Becton Dickinson).

### 2.17 Magnetic separation of the CD133^+^/CD44^+^ cells

Detached cells (1 × 10^7^) were incubated with the microbead‐conjugated CD133 antibodies before being magnetically separated using a CD133 Cell Isolation Kit (Miltenyi Biotec). The collected CD133^+^ cells were next incubated by the microbead‐conjugated CD44 antibodies and then subjected to magnetic separation using a CD44 Cell Isolation Kit (Miltenyi Biotec) to isolate the CD133^+^/CD44^+^ cells.

## 3 Results

### 3.1 LRH‐1 is a direct target of GATA6

**Figure 1:**
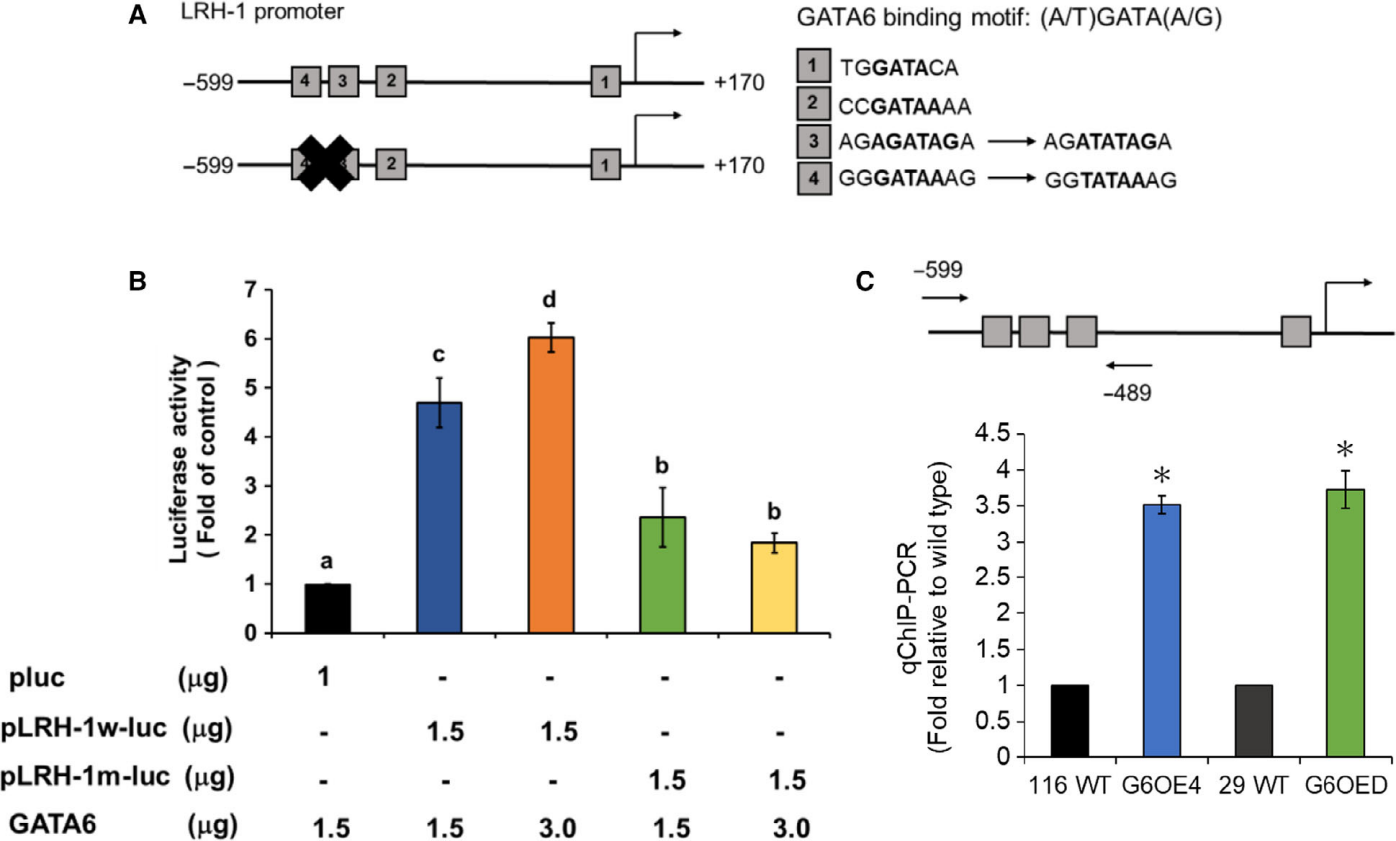
GATA6 can directly bind and activate the *LRH‐1* promoter. (A) Schematic showing of the promoter region of human *LRH‐1* gene with four putative GATA6‐binding sites (boxes with their respective sequences) as well as the mutations introduced on two of them. (B) The reporter plasmids carrying a luciferase gene without (pluc) or with the upstream insertion of either the wild‐type (pLRH‐1w‐luc) or mutated (pLRH‐1m‐luc) LRH‐1 promoter were transiently cotransfected with different combinations of an empty (pBabe) or a GATA6‐expression (GATA6) vector as well as 1 mg pCMV‐LacZ (for normalizing transfection efficiency) into 293T cells. Twenty‐four hours later, 10 µg of total lysates was used, respectively, for analyzing the activities of luciferase and β‐glucosidase. Data (mean ± SD, *N* = 3) were analyzed by one‐way ANOVA with the LSD *post hoc* test, and the different characters represent different levels of significance (*P* < 0.05). (C) ChIP assays were carried out as described in the ‘Materials and methods’ with the primer annealing sites (arrows) shown in the top drawing. ^＊^ *P* < 0.05 when compared with the corresponding the wild‐type cells by Student's *t*‐test.

Because *LRH‐1* has been proposed as a potential target of GATA6 (Sulahian *et al.*, 2013) plus our recent work found that knockdown of *LRH‐1* expression could decrease the stemness in the GATA6‐overexpressing human CRC cells (Lai *et al.*, 2020), we first analyzed whether *LRH‐1* is a direct target gene of GATA6. Since four putative GATA6‐binding sites were identified in the promoter region (−599 to +170) of *LRH‐1* (Fig. 1A), we constructed two reporter plasmids with insertion of either the wild‐type LRH‐1 promoter or the one containing two mutated GATA6‐binding sites upstream to the luciferase gene (designated as pLRH‐1wluc and pLRH‐1mluc in Fig. 1A). While a dose‐dependent increase of the luciferase activity was detected in cells when pLRH‐1wluc was cotransfected with different amounts of GATA6 expression plasmid, a GATA6‐independent reduced stimulation of the reporter activity was observed in cells cotransfected with pLRH‐1mluc and GATA6 expression plasmid (Fig. 1B). These results suggested that the two furthest putative GATA6‐binding sites in the *LRH‐1* promoter were mainly responsible for the activation of this promoter by GATA6. Quantitative chromatin immunoprecipitation (qChIP) was then performed to confirm this speculation. As expected, a much higher binding of GATA6 to the *LRH‐1* promoter region containing these binding sites was detected in the GATA6‐overexpressing clones established previously from both HCT‐116 (G6OE4) and HT‐29 (G6OED) human CRC lines (Fig. 1C).

### 3.2 Upregulation of LRH‐1 in human CRC cells increases their stemness properties

**Figure 2:**
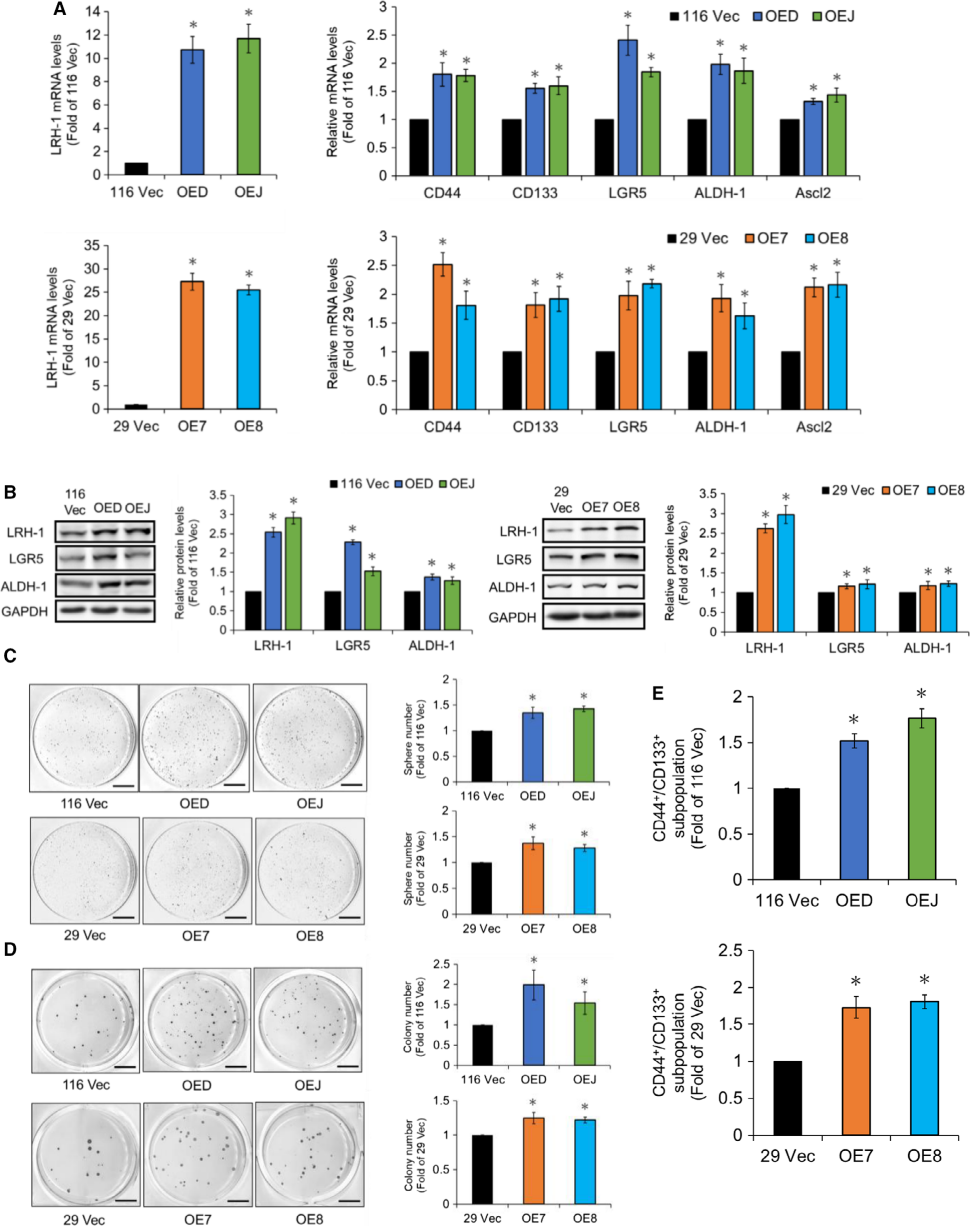
LRH‐1 overexpression increases various stemness properties in HCT‐116 and HT‐29 cells. (A) The mRNA levels of *LRH‐1* (left) as well as CRCSC markers such as *CD44*, *CD133*, *LGR5*, *ALDH‐1,* and *Ascl2* (right) in the vector control (116 Vec) and the LRH‐1‐overexpressing HCT‐116 (OED and OEJ) (top) as well as the vector control (29 Vec) and the LRH‐1‐overexpressing HT‐29 (OE7 and OE8) (bottom) clones were analyzed by RT‐qPCR. (B) Total lysates (30 µg) prepared from the 116 Vec, OED, and OEJ clones (left) as well as the 29 Vec, OE7, and OE8 clones (right) were subjected to immunoblot analysis using antibodies against the indicated proteins. GAPDH signals were used as loading controls. (C) Cells from three HCT‐116 clones (1 × 10^4^/well, upper) and three HT‐29 clones (5 × 10^4^/well, lower) were cultured, respectively, in defined media for 20 days. Spheres stained by MTT were scanned, and their numbers were counted by metamorph software. Scale bar, 0.7 cm. (D) Cells from three HCT‐116 clones (1 × 10^3^/well, upper) and three HT‐29 clones (1 × 10^4^/well, lower) were cultured in 0.3% agar on top of 0.6% agar for 20 days. Colonies stained by MTT were scanned and their numbers were counted by metamorph software. Scale bar, 0.7 cm. (E) Flow cytometry was applied to examine the CD133^+^/CD44^+^ subpopulations in the 116 Vec, OED, and OEJ clones (upper) as well as the 29 Vec, OE7, and OE8 clones (lower), respectively. Data are mean ± SD from three independent experiments. *P < 0.05 compared with those of the corresponding vector‐control clones by Student's *t*‐test.

Having found that *LRH‐1* is a direct target of GATA6, we next examined whether upregulation of *LRH‐1* could enhance the stemness in HCT‐116 and HT‐29 cells. Stable LRH‐1‐overexpressing clones were established respectively from HCT‐116 (OED and OEJ) and HT‐29 (OE7 and OE8) cells as described in the ‘Materials and methods’. RT‐qPCR was then performed to measure the mRNA levels of *LRH‐1* as well as several CRCSC markers such as *CD44*, *CD133*, *LGR5*, *ALDH‐1,* and *Ascl2*. As shown in Fig. 2A, the mRNA levels of the aforementioned genes were all significantly increased in these clones. Increased protein levels of LRH‐1 as well as two CRCSC markers LGR5 and ALDH‐1 in these clones were subsequently verified by immunoblotting (Fig. 2B). Increased expression of several ESC markers (i.e., *Oct4, Klf4, Nanog, and Sox2*) in the aforementioned LRH‐1‐overexpressing clones was further demonstrated by RT‐qPCR and western blot analyses (Fig. S1). To determine whether the self‐renewal abilities of human CRC cells were enhanced by LRH‐1 upregulation, sphere‐forming assays were conducted. To no surprise, the numbers of spheres grown from the LRH‐1‐overexpressing clones were significantly higher than their respective vector‐control clones (Fig. 2C). The anchorage‐independent growth abilities of these clones were then assessed by the soft agar colony formation assay. As expected, the anchorage‐independent growth abilities of the LRH‐1‐overexpressing clones were also drastically increased (Fig. 2D). Finally, flow cytometric analysis showed that the CD133^+^/CD44^+^ subpopulations were substantially increased in the LRH‐1 overexpressing HCT‐116 and HT‐29 clones (Fig. 2E).

### 3.3 HIF‐1α activity is elevated in the LRH‐1‐overexpressing clones

**Figure 3:**
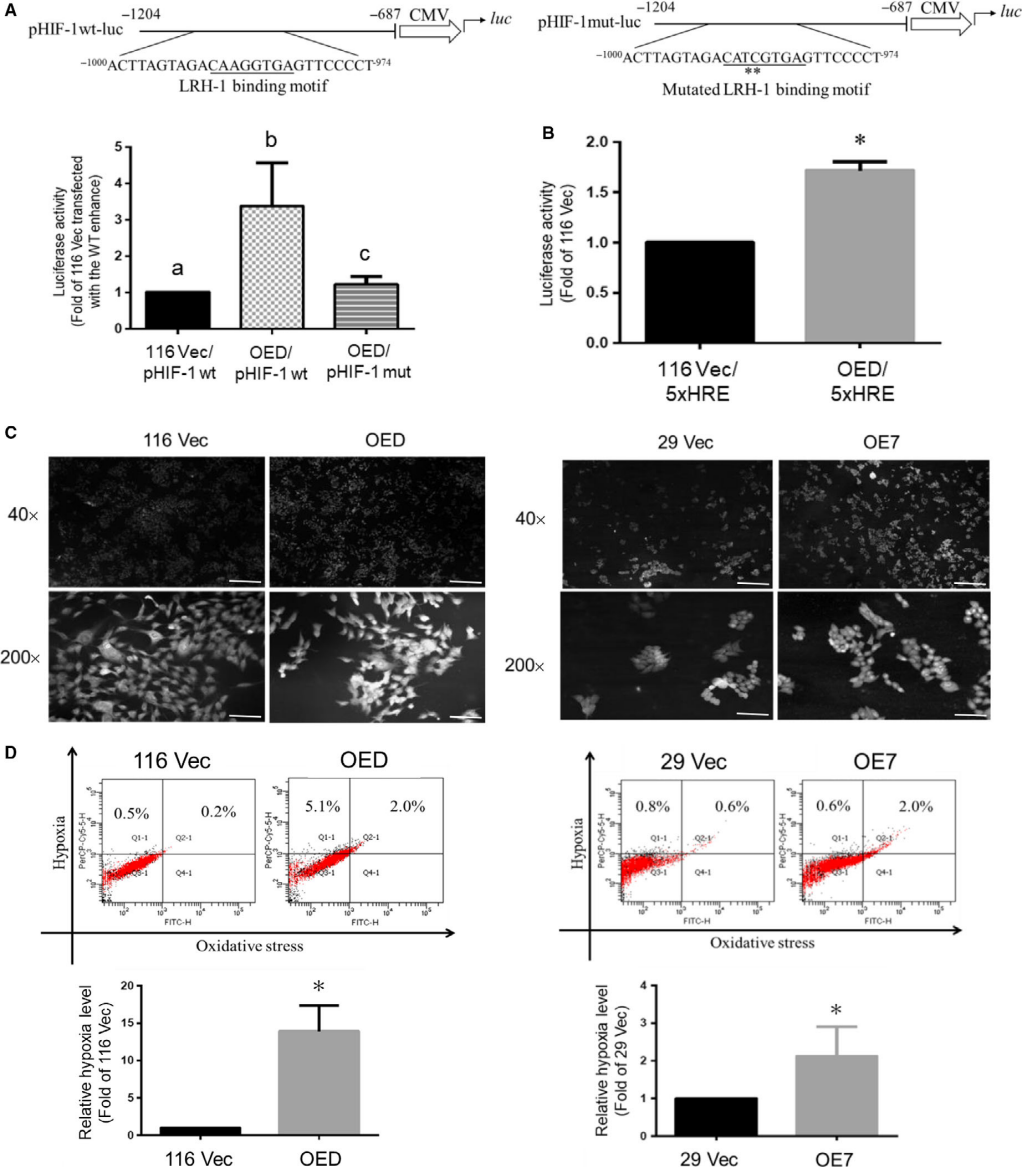
*HIF‐1α* promoter region contains an enhancer which can be activated directly by LRH‐1 and higher HIF‐1α activity as well as hypoxia are detected in the LRH‐1‐overexpressing clones. (A) Total lysates (5 µg) prepared, respectively, from the 116 Vec and OED cells after they were transfected with the plasmids carrying a luciferase gene driven by a *HIF‐1α* enhancer containing either a wild‐type (pHIF‐1wt‐luc) or a mutated (pHIF‐1mut‐luc) LRH‐1 binding motif (top drawings) were subjected to the reporter assays. Transfection efficiency was normalized with the activities of β‐glycosidase. Data (mean ± SD, *N* = 3) were analyzed by one‐way ANOVA with the LSD *post hoc* test and different characters represent different levels of significance (*P* < 0.05). (B) Total lysates (5 µg) prepared, respectively, from the 116 Vec and OED cells after they were cotransfected with p5xHRE‐luc (1.5 µg) and pCMV‐lacZ (1 µg) were subjected to reporter assay to determine their respective luciferase activity. 116 Vec and OED (left) as well as 29 Vec and OE7 (right) cells were seeded onto microscopic slides. Two days later, the intracellular hypoxia status was detected by (C) fluorescence microscopy and (D) flow cytometry after cells were stained by the intracellular hypoxia reagent for 30 min. The numbers shown on the left of (C) are fold of magnification. Scale bar, 100 μm. Data are mean ± SD of three independent experiments. *P < 0.05 compared with that of the corresponding vector‐control clones by Student's *t*‐test.

Having demonstrated that upregulation of LRH‐1 could increase the stemness properties in human CRC cells, next we wanted to identify the mediator(s) of the stemness‐stimulating effect of LRH‐1. Among all the potential candidates predicted earlier by others (Simandi *et al.*, 2013), hypoxia‐inducible factor‐1α (HIF‐1α) was the most interesting one because it is known to play a crucial role in the maintenance of stemness in normal as well as CSCs (Keith and Simon, 2007; Santoyo‐Ramos *et al.*, 2014; Soeda *et al.*, 2009). To verify whether *HIF‐1α *is a direct target gene of LRH‐1, we search its promoter region and found one putative binding site for this transcription factor (Fig. 3A). Hence, we constructed two reporter plasmids with upstream insertion of the HIF‐1α promoter region (−1204 to −687) containing either a wild‐type (pHIF‐1wt‐luc) or a mutated (pHIF‐1mut‐luc) LRH‐1‐binding site (Fig. 3A) to the luciferase gene whose expression is controlled by a minimal CMV promoter. As can be seen in Fig. 3A, the luciferase activity was significantly increased when pHIF‐1wt‐luc was transfected into the LRH‐1‐overexpressing HCT‐116 clone (OED). Conversely, the reporter activity was not changed when pHIF‐1mut‐luc was transfected into similar cells. To further prove that HIF‐1α activity was indeed higher in the LRH‐1‐overexpressing HCT‐116 clones, a luciferase reporter whose expression is driven by an enhancer containing five repeats of hypoxia response element (5xHRE‐luc) was transfected into 116 Vec and OED clones, respectively. As expected, significantly higher luciferase activity was detected in the OED clone (Fig. 3B). Since hypoxia and/or high intracellular ROS level are essential for the maintenance of HIF‐1α activity, we measured the intracellular hypoxia and oxidative stress levels simultaneously in the vector‐control and LRH‐1‐overexpressing clones using a commercial kit. As can be seen, much stronger immunofluorescence intensities were observed in both LRH‐1‐overexpressing HCT‐116 (OED) and HT‐29 (OE7) clones (Fig. 3C). In addition, flow cytometry was applied to quantify both the intracellular hypoxia and oxidative stress levels. Interestingly, over 10‐fold and approximately twofold increases of the intracellular hypoxia levels were detected, respectively, in the LRH‐1‐overexpressing HCT‐116 and HT‐29 clones (Fig. 3D).

### 3.4 Increased glycolysis in the LRH‐1‐overexpressing clones is likely mediated by HIF‐1α

**Figure 4:**
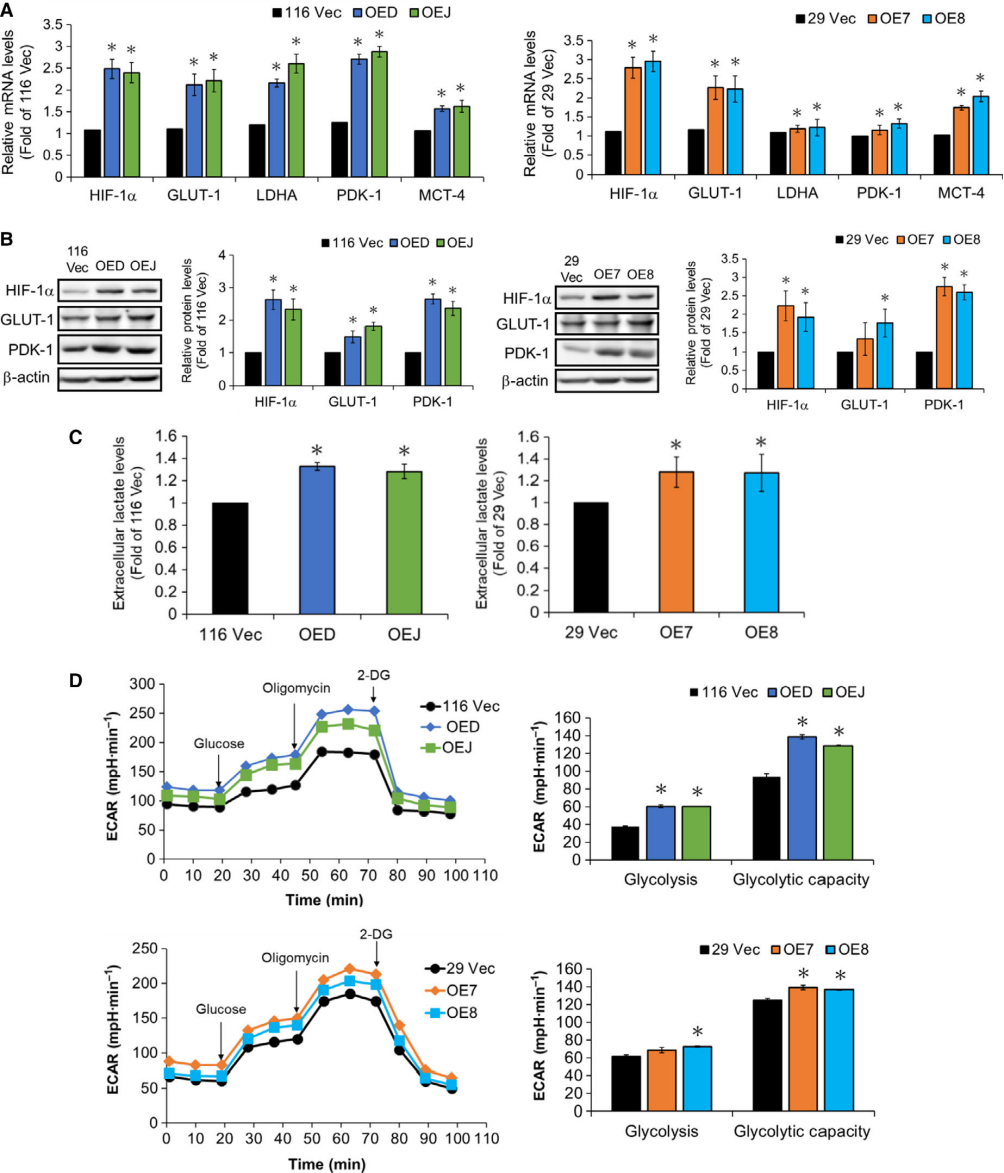
Analyses of the expression levels of HIF‐1α and several of its downstream target genes as well as the glycolytic functions in the LRH‐1‐overexpressing clones. (A) The mRNA levels of *HIF‐1α* as well as *GLUT‐1*, *LDHA*, *PDK‐1*, and *MCT‐4* in the 116 Vec, OED, and OEJ clones (left) as well as the 29 Vec, OE7, and OE8 clones (right) were analyzed, respectively, by RT‐qPCR. (B) Total lysates (30 µg) prepared from the aforementioned clones were subjected to immunoblot analysis using antibodies against HIF‐1α, GLUT‐1, and PDK‐1, respectively. Beta‐actin signals were used as loading controls. (C) The culture media were collected from the aforementioned clones after cells being seeded for 48 h, and their lactate levels were measured as described in the ‘Materials and methods’. (D) Glycolytic stress tests were performed using the Seahorse XF bioanalyzer to measure the glycolytic capacities of the 116 Vec, OED, and OEJ clones (top) as well as the 29 Vec, OE7, and OE8 clones (bottom). Data are mean ± SD of three independent experiments. *P < 0.05 compared with those of the corresponding vector‐control clones by Student's *t*‐test.

Since HIF‐1α activity was markedly increased in the LRH‐1‐overexpressing clones, we subsequently assessed whether the expression levels of HIF‐1α as well as some of its downstream target genes including *Glut‐1* (glucose transporter 1), *LDHA* (lactate dehydrogenase A), *PDK‐1* (pyruvate dehydrogenase kinase 1), and *MCT‐4* (monocarboxylate transporter‐4) were also elevated in them. To no surprise, the mRNA levels of all the aforementioned genes were markedly increased in the LRH‐1‐overexpressing HCT‐116 and HT‐29 clones (Fig. 4A). Immunoblotting was subsequently conducted to confirm that HIF‐1α, GLUT‐1, and PDK‐1 protein levels were increased (Fig. 4B). Since HIF‐1α has been shown to promote glycolysis (Semenza, 2010), we next measured the extra‐ and intracellular levels of lactate, the end product of glycolysis, in the LRH‐1‐overexpressing HCT‐116 and HT‐29 clones. As expected, both the extra‐ (Fig. 4C) and intra‐ (Fig. S2A) cellular lactate levels were significantly increased in these clones. Conversely, a markedly reduction of lactate levels was found in these cells after they were treated with oxamate, a LDHA inhibitor (Fig. S2B,C). The glycolytic activity in the aforementioned clones was further examined by Seahorse Extracellular Flux XF‐24 analyzer. Using ECAR as a readout for glycolysis, our results showed that both the glycolysis and glycolytic capacity were markedly elevated in the LRH‐1‐overexpressing clones (Fig. 4C). Together, these findings suggested that the increased glycolysis in the LRH‐1‐overexpressing human CRC cells might be accounted by an upregulated HIF‐1α activity.

### 3.5 Increased ROS production and mitochondrial respiration are also detected in the LRH‐1‐overexpressing clones

**Figure 5:**
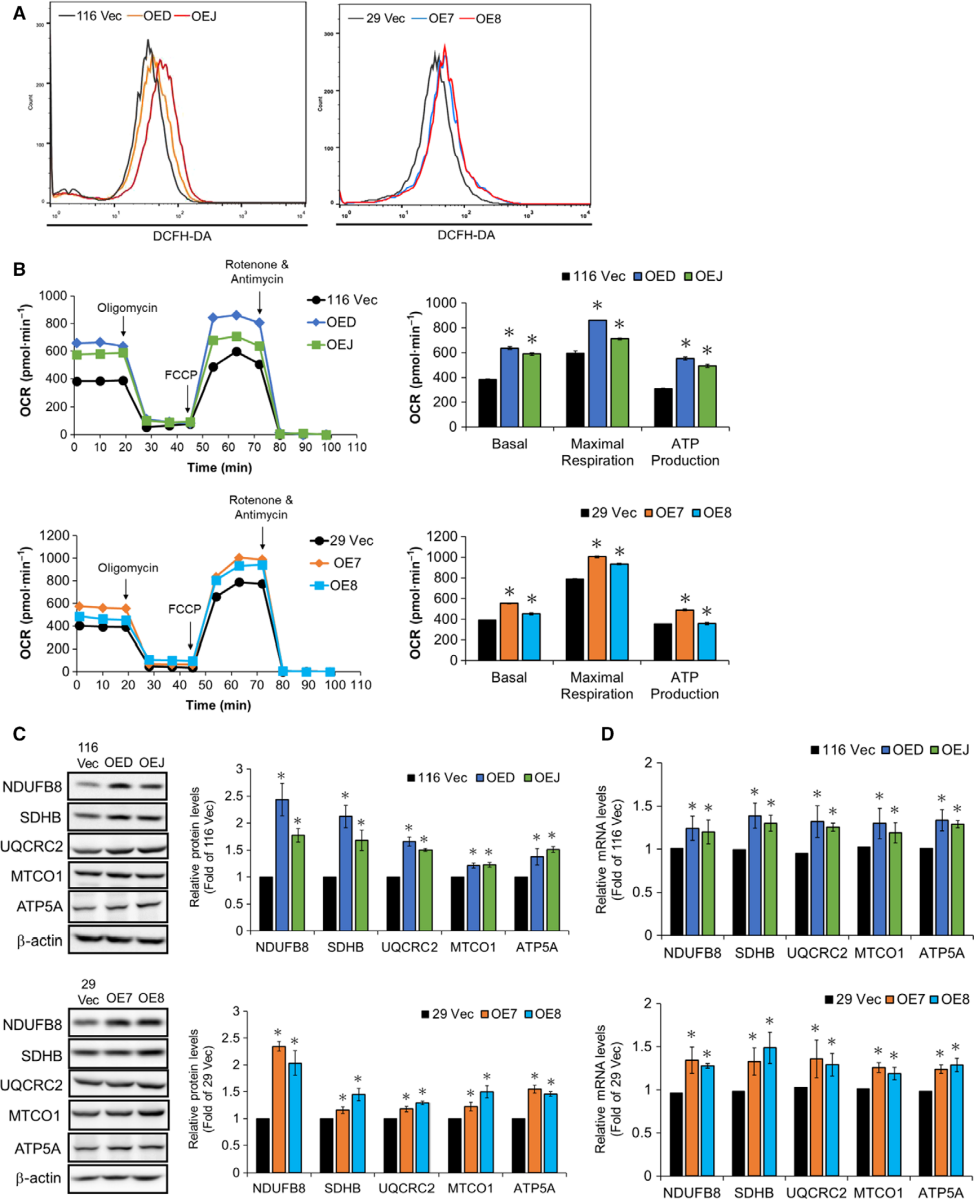
Mitochondrial functions are elevated in the LRH‐1‐overexpressing clones. (A) The intracellular ROS levels in the 116 Vec, OED, and OEJ clones (left) as well as the 29 Vec, OE7, and OE8 clones (right) were detected by flow cytometry after they were stained with 1 mm DCFH‐DA. (B) Mitochondrial respiration tests were performed using the Seahorse XF bioanalyzer to measure the basal and maximal respiration as well as ATP production of three HCT‐116 (top) and three HT‐29 (bottom) clones, respectively. (C) Total lysates (30 µg) prepared from the 116 Vec, OED, and OEJ clones (upper) as well as the 29 Vec, OE7, and OE8 clones (lower) were subjected to immunoblot analysis using antibodies against specific subunits for each ETC complex (e.g., NDUFB8, SDHB, UQCRC2, MTCO1, and ATP5A for complexes I, II, III, IV, and V, respectively) as probes, respectively. Beta‐actin signals were used as loading controls. (D) The mRNA levels of the aforementioned subunits for each ETC complex in the LRH‐1‐overexpressing HCT‐116 (top) and HT‐29 (bottom) clones were analyzed by RT‐qPCR. Data are mean ± SD of three independent experiments. *P < 0.05 compared with those of the corresponding vector‐control clones by Student's *t*‐test.

Since markedly increased protein levels of PDK‐1, which is known to inhibit pyruvate dehydrogenase (PDH), causing the shunting of pyruvate from the mitochondria and subsequently attenuating mitochondrial respiration and ROS production (Papandreou *et al.*, 2006), were found in the LRH‐1‐overexpressing clones, we next examined the ROS levels in the 116 Vec, OED, and OEJ clones as well as the 29Vec, OE7, and OE8 clones by flow cytometry. Strikingly, the ROS levels were much higher in the LRH‐1‐overexpressing clones derived from both HCT‐116 and HT‐29 cells (Fig. 5A). In accordance, the OCR, as a measure of cellular respiration and mitochondrial function, was also increased robustly in these clones (Fig. 5B). Immunoblot and RT‐qPCR analyses were subsequently applied to examine the expression levels of the components of different ETC (electron transport chain) complexes including NDUFB8 (complex I), SDHB (complex II), UQCRC2 (complex III), MTCO1 (complex IV), and ATP5A (complex V). As expected, both protein (Fig. 5C) and mRNA (Fig. 5D) levels of these components were all increased significantly in the LRH‐1‐overexpressing clones. Collectively, our results suggested that there might be two different populations (i.e., glycolytic and oxidative) present in these clones.

### 3.6 Lactate‐mediated upregulation of PGC‐1α and MCT‐1 is crucial for the emergence of the oxidative populations in the LRH‐1‐overexpressing clones

**Figure 6:**
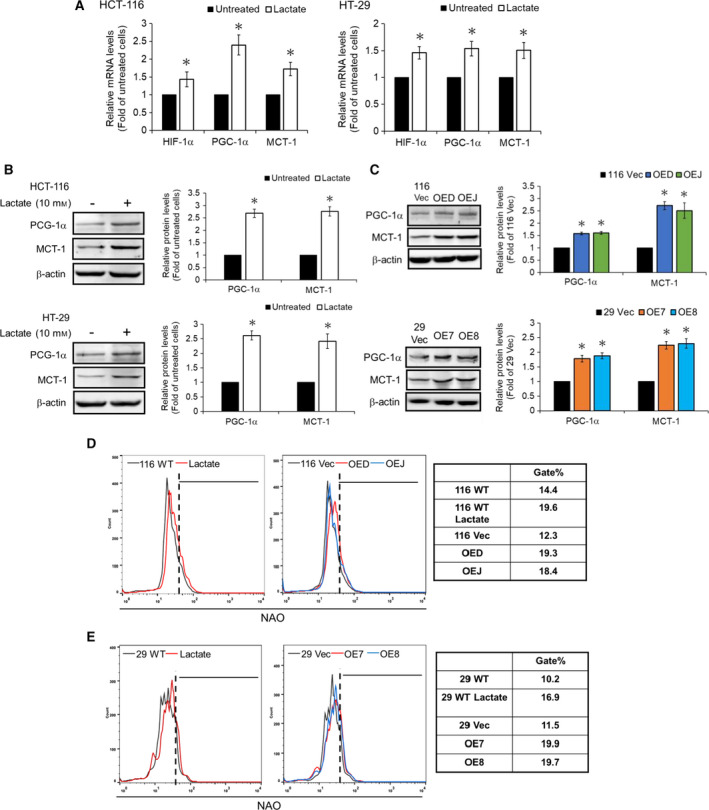
Lactate is a critical mediator to promote the mitochondrial biogenesis in human colon cancer cells. (A) The wild‐type HCT‐119 (left) and HT‐29 (right) cells were treated without or with 10 mm lactate for 48 hr before the mRNA levels of HIF‐1α, PGC‐1α, and MCT‐1 were analyzed by RT‐qPCR. (B) Total lysates (30 µg) prepared from the wild‐type HCT‐119 (upper) and HT‐29 (lower) cells after similar treatments were subjected to immunoblot analysis using antibodies against PGC‐1α and MCT‐1 as probes, respectively. Beta‐actin signals were used as loading controls. Data are mean ± SD of three independent experiments. *P < 0.05 compared with those of the corresponding wild‐type cells by Student's *t*‐test. (C) Total lysates (30 µg) prepared from the 116 Vec, OED, and OEJ clones (upper) as well as the 29 Vec, OE7, and OE8 clones (lower) were subjected to immunoblot analysis using antibodies against PGC‐1α and MCT‐1 as probes, respectively. Beta‐actin signals were used as loading controls. Data are mean ± SD of three independent experiments. *P < 0.05 compared with those of the corresponding vector‐control clones by Student's *t*‐test. The mitochondrial mass of (D) the wild‐type HCT‐116 cells before and after treatment with lactate (left) as well as the 116 Vec, OED, and OEJ clones (middle), and (E) the wild‐type HT‐29 cells before and after treatment with lactate (left) as well as the 29 Vec, OE7, and OE8 clones (middle) were measured by flow cytometry after they were stained with 100 nm NAO. The gate population (right) was measured by flowjo V10 software.

To understand how the oxidative population emerged in the LRH‐1‐overexpressing clones, we turned our attention to lactate since previous studies have shown that this metabolite could not only stabilize HIF‐1α (Sonveaux *et al.*, 2012) but also increase the mRNA levels of peroxisome proliferator‐activated receptor γ coactivator‐1α (PGC‐1α) (Kitaoka *et al.*, 2016) which plays a key role in mitochondrial biogenesis and energy metabolism (Wu *et al.*, 1999). As can be seen, both the mRNA (Fig. 6A) and protein (Fig. 6B) levels of PGC‐1α and MCT‐1 in the wild‐type HCT‐116 and HT‐29 cells were robustly increased by the treatment with lactate (10 mm). In addition, markedly increased PGC‐1α and MCT‐1 protein levels were also detected in the LRH‐1‐overexpressing clones derived from both these cells (Fig. 6C) which might account for our earlier observations of the significantly elevated expression levels of the components of various ETC complexes in them (Fig. 5C,D). NAO staining was subsequently used to confirm the significant increases in mitochondrial mass in the wild‐type HCT‐116 and HT‐29 cells treated with exogenous lactate as well as in the LRH‐1‐overexpressing clones derived from them (Fig. 6D,E). Together, our results strongly suggested that lactate‐mediated upregulation of PGC‐1α and MCT‐1 is crucial for the emergence of the oxidative populations in the LRH‐1‐overexpressing human CRC clones.

### 3.7 Both glycolysis and oxidative phosphorylation are important for the self‐renewal of human CRCSCs

**Figure 7:**
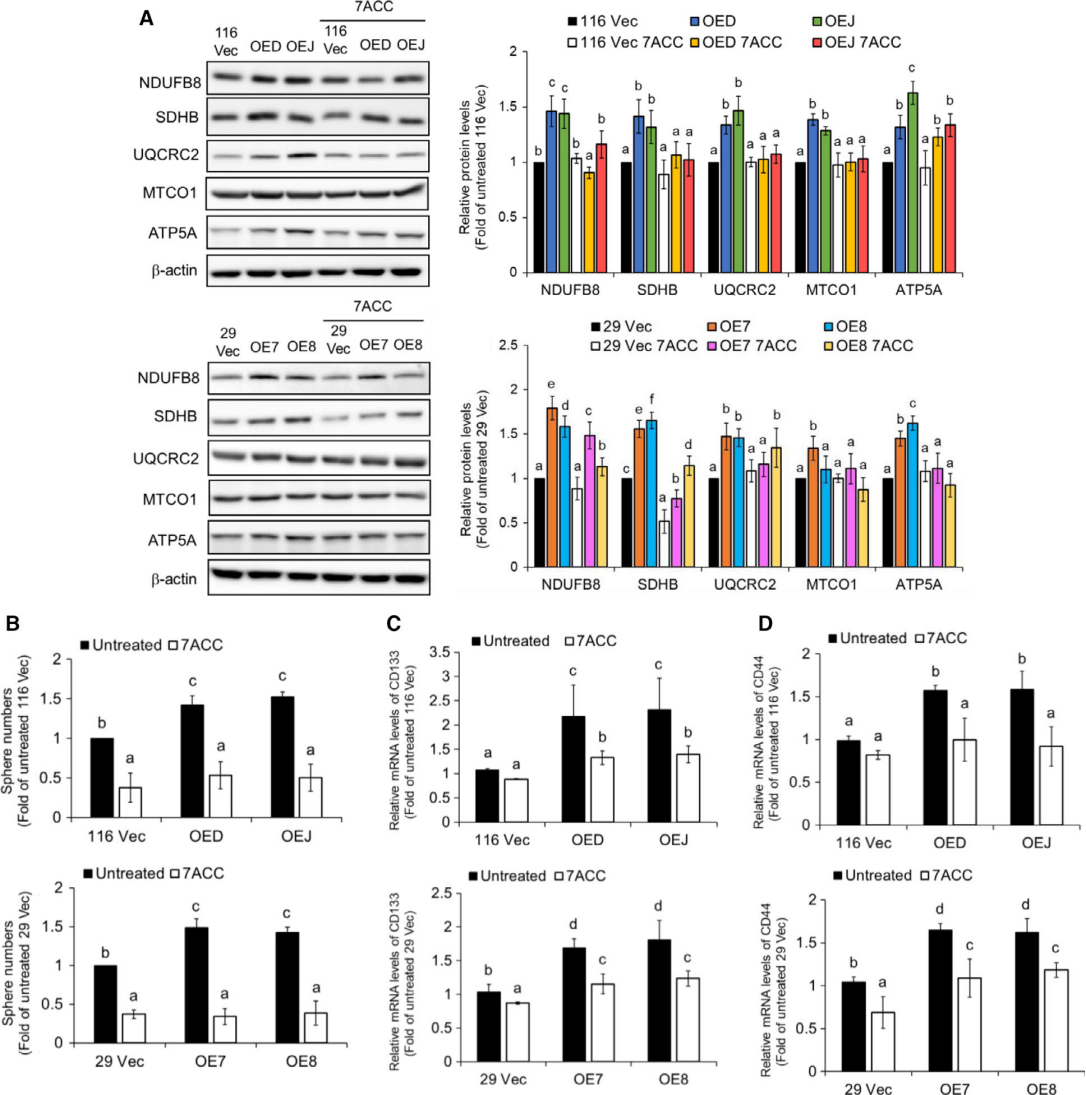
Lactate uptake is crucial for the increase of the subunits of various mitochondrial ETC complexes and the stemness in human colon cancer cells. (A) Total lysates (30 µg) prepared from the 116 Vec, OED, and OEJ clones (upper) as well as the 29 Vec, OE7, and OE8 clones (lower) after being treated without or with 20 nm 7ACC for 48 h were subjected to immunoblot analysis using antibodies against the aforementioned subunits of various ETC complexes as probes, respectively. Beta‐actin signals were used as loading controls. (B) Cells from three HCT‐116 clones (upper) and three HT‐29 clones (lower) were cultured, respectively, in defined media supplemented without or with 20 nm 7ACC for 20 days. Spheres stained by MTT were scanned, and their numbers were counted by MetaMorph software. The mRNA levels of CD133 (C) and CD44 (D) in three HCT‐116 (upper) and three HT‐29 (lower) clones were analyzed by RT‐qPCR after they were treated without or with 20 nm 7ACC for 48 h. Data (mean ± SD, *N* = 3) were analyzed by one‐way ANOVA with the LSD *post hoc* test, and different characters represent different levels of significance (*P* < 0.05).

Since we found that the lactate could enhance the mitochondrial biogenesis in human CRC cells, we then evaluated the effects of a selective MCT‐1 inhibitor, 7ACC (7‐aminocarboxycoumarin), on the expression levels of the components of various ETC complexes in the LRH‐1‐overexpressing clones by immunoblotting. As shown in Fig. 7A, the significant increases in the protein levels of NDUFB8, SDHB, UQCRC2, MTCO1, and ATP5A in these clones were mostly reduced by 7ACC treatment. In good agreement, marked decreases in the intracellular ROS levels were also detected in the LRH‐1‐overexpressing but not in the vector‐control clones treated with 7ACC (Fig. S3A,B). Furthermore, 7ACC treatment caused significant reductions in the sphere‐forming ability (Fig. 7B) as well as the mRNA levels of CD133 (Fig. 7C) and CD44 (Fig. 7D) in these clones. Collectively, our findings suggested that lactate secreted from the glycolytic/hypoxic population could be uptaken by the oxidative population via MCT‐1 which in turn stimulated the mitochondrial biogenesis, oxidative phosphorylation (OXPHOS), and stemness by activating PGC‐1α.

The respective contributions of glycolysis and OXPHOS in maintaining the stemness of human CRC cells were next assessed by conducting the sphere formation assays in the absence or presence of oxamate and rotenone (a mitochondrial ETC complex I inhibitor), respectively. To our surprise, marked decreases of the sphere number were detected in both the vector‐control and the LRH‐1‐overexpressing clones after their glycolysis and OXPHOS being suppressed, respectively, by oxamate and rotenone (Fig. S4A). In the meantime, since ROS signaling could regulate the homeostasis of stem cells (Wang *et al.*, 2013) plus significantly increased ROS levels were observed in the LRH‐1 overexpressing clones, we analyzed the role of ROS in the self‐renewal of these cells by treating them without or with a potent ROS scavenger, NAC (*N*‐acetylcysteine). Strikingly, dramatic decreases in the self‐renewal ability were found in the LRH‐1‐overexpressing as well as the vector‐control clones treated with NAC (Fig. S4B). To confirm the stimulatory role of ROS in the self‐renewal ability of human CRC cells, sphere formation assays of the aforementioned clones were carried out in the absence and presence of different doses of another antioxidant, ascorbic acid. In good agreement, a dose‐dependent reduction in the sphere‐forming ability of these cells was detected (Fig. S4C). Together, these results strongly suggested that both glycolysis and OXPHOS are critical for maintaining certain stemness properties in human CRCSCs and ROS is an essential mediator in the latter.

### 3.8 Human CRCSCs are a predominantly OXPHOS metabolic phenotype

**Figure 8:**
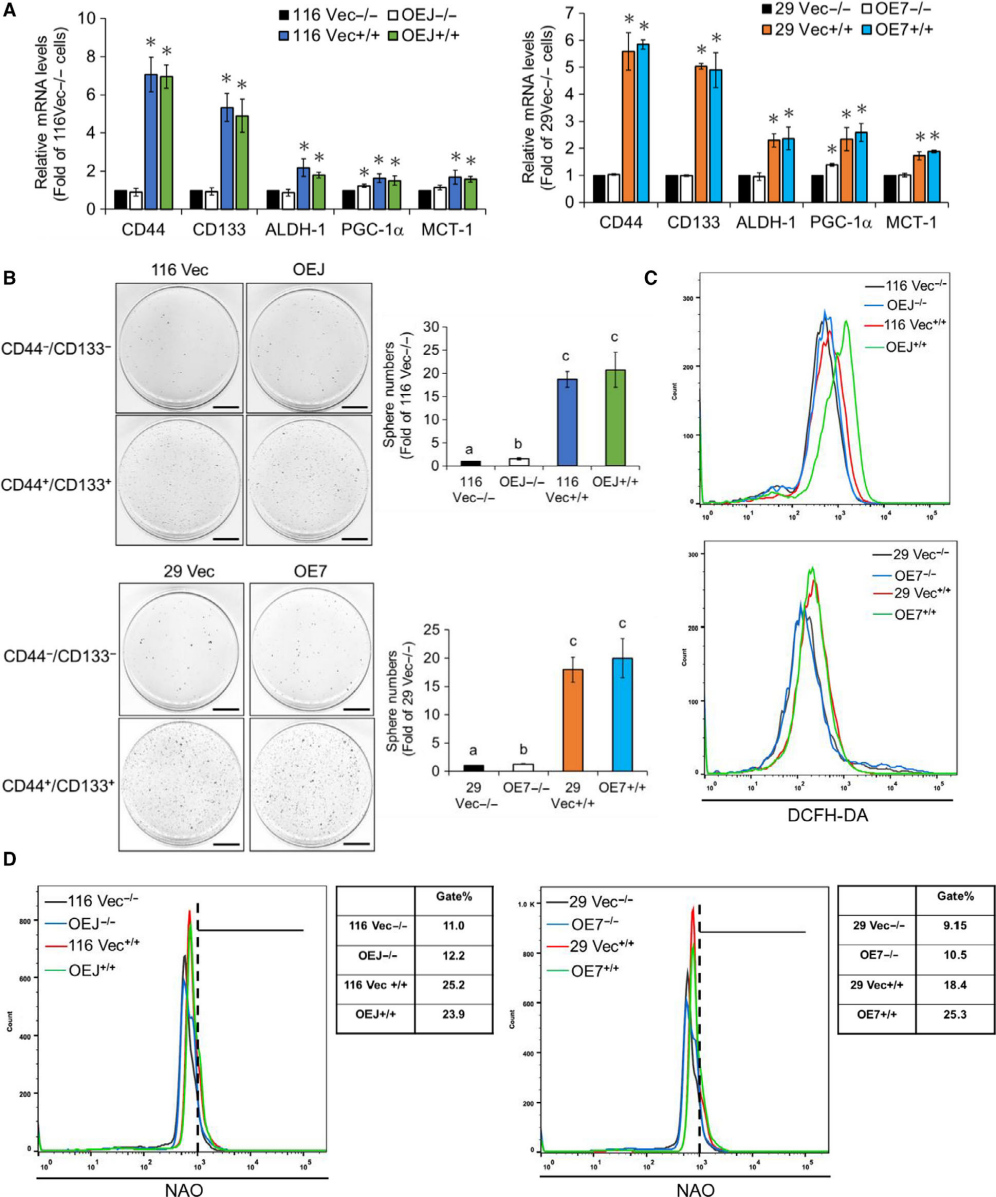
Oxidative phosphorylation is the main metabolism in the CD133+/CD44+ stem‐like human colon cancer cells. (A) The mRNA levels of CD44, CD133, ALDH‐1, PGC‐1α, and MCT‐1 in the CD133^−^/CD44^−^ and CD133^+^/CD44^+^ cells isolated, respectively, from the 116 Vec (−/− & +/+) and OEJ (−/− & +/+) clones as well as the 29 Vec (−/− & +/+) and OE7 (−/−& +/+) clones by the magnetic separation system were analyzed by RT‐qPCR. Data are mean ± SD of three independent experiments. *P < 0.05 compared with those of the corresponding Vec−/− cells by Student's *t*‐test. (B) The 116 Vec −/− & +/+ and OEJ −/− & +/+ cells as well as the 29 Vec −/− & +/+ and OE7 −/− & +/+ cells were cultured, respectively, in defined media for 20 days. Spheres stained by MTT were scanned, and their numbers were counted by metamorph software. Data (mean ± SD, *N* = 3) were analyzed by one‐way ANOVA with the LSD *post hoc* test, and different characters represent different levels of significance (*P* < 0.05). (C) The ROS levels in the 116 Vec −/− & +/+ and OEJ −/− & +/+ cells (upper) as well as the 29 Vec −/− & +/+ and OE7 −/− & +/+ cells (lower) were analyzed by flow cytometry after they were stained with 1 mm DCFH‐DA. (D) The mitochondrial mass in the 116 Vec −/− & +/+ and OEJ −/− & +/+ cells (left) as well as the 29 Vec −/− & +/+ and OE7 −/− & +/+ cells (right) was measured by flow cytometry after they were stained with 100 nm NAO. The gate population was measured by flowjo V10 software.

Having found that both glycolysis and OXPHOS are critical for human CRCSCs, we liked to find out which metabolic pathway is prevailed in these cells since controversial results have previously been reported in other types of CSCs (Peiris‐Pagès *et al.*, 2016). For this purpose, two widely used CSC markers, CD133 (O'Brien *et al.*, 2006; Ricci‐Vitiani *et al.*, 2006) and CD44 (Dalerba *et al.*, 2007) were chosen to isolate human CRCSCs from some of the aforementioned clones. After magnetic separation, mRNA levels of three stemness markers (i.e., CD133, CD44, and ALDH‐1) as well as PGC‐1α and MCT‐1 in the CD133^+^/ CD44^+^ (double‐positive) SC‐like population and the CD133^‐^/CD44^‐^ (double‐negative) non‐SC population were analyzed, respectively, by RT‐qPCR. As expected, the mRNA levels of three CRCSC markers, PGC‐1α and MCT‐1 were all significantly higher in the double‐positive SC‐like populations (Fig. 8A). Sphere formation assays were subsequently conducted to analyze the self‐renewal ability of these different populations. To no surprise, much higher self‐renewal ability was observed in the CD133^+^/CD44^+^ cells isolated from both the vector‐control and the LRH‐1‐overexpressing clones. Interestingly, this stemness property in the double‐negative non‐SC population isolated from the LRH‐1‐overexpressing clones was significantly stronger than that from the vector‐control clones (Fig. 8B). After confirming that the double‐positive population indeed had higher stemness, we next assessed its main metabolic pathway. Flow cytometry was applied to first compare the intracellular ROS levels in different populations. As shown in Fig. 8C, the ROS levels were dramatically elevated in the CD133^+^/CD44^+^ cells regardless of their sources. In addition, significant increases in the mitochondrial mass were also found in these populations (Fig. 8D). Together, our results indicated that the SC‐like population in human CRC cells relies more on the mitochondrial respiration.

## 4 Discussion

Metastatic colorectal cancer remains one of the deadliest malignancies worldwide with a 5‐year survival rate of 10% (Haggar and Boushey, 2009) which could be accounted mainly by the presence of CRCSCs (Zeki *et al*., 2011). We have previously shown that GATA6 robustly enhances the stemness properties in HCT‐116 and HT‐29 human CRC cells (Lai *et al.*, 2020). In this work, we aimed to identify the downstream target(s) of GATA6 responsible for increasing the stemness of human CRC cells as well as dissect the underlying mechanisms. Regarding the potential target(s) of GATA6 that might be crucial in stimulating CRCSCs, liver receptor homolog‐1 (LRH‐1) is of particular interest because this transcription factor has been reported to play a role in CSC stemness and EMT in pancreatic cancer (Luo *et al.*, 2017). In addition, our previous results showed that knockdown of *LRH‐1* expression in GATA6‐overexpressing HCT‐116 and HT‐29 cells drastically reduced their self‐renewal abilities (Lai *et al.*, 2020). Furthermore, LRH‐1 could act as a coactivator of the β‐catenin signaling pathway to promote intestinal cell renewal (Botrugno *et al.*, 2004). Another earlier study even demonstrated that LRH‐1 could replace Oct4 in the derivation of iPSCs from mouse somatic cells as well as enhance reprogramming efficiency (Heng *et al.*, 2010). Collectively, these findings suggest that LRH‐1 might play a crucial role in upregulating the stemness properties of CRCSCs.

In this study, reporter assay and ChIP were first used to confirm that *LRH‐1* was indeed a downstream target of GATA6 (Fig. 1). Subsequently, LRH‐1‐overexpressing stable clones were established, respectively, from HCT‐116 and HT‐29 cells and marked increases in several prominent stemness properties (e.g., elevated expression levels of various CRCSC markers, stronger self‐renewal as well as anchorage‐independent growth abilities, and higher CD133^+^/CD44^+^ subpopulations) were detected in these clones (Fig. 2 and Fig. S1). Together, these results robustly suggest that *LRH‐1* upregulation induced by GATA6 can promote the stemness properties in human CRC cells.

Hypoxia is a common feature of solid tumors and tumor cells often adapt to this stressful condition by activating hypoxia‐inducible factor 1 (HIF‐1) (Semenza, 2001), a heterodimeric transcription factor composed of HIF‐1α, an oxygen sensitive subunit, and HIF‐1β, a constitutively expressed subunit (Wang *et al.*, 1995). So far, a number of genes including those encoding proteins that participate in angiogenesis, glucose metabolism, cell proliferation, and survival have been identified to be activated by HIF‐1α (Semenza, 2001). Interestingly, silencing HIF‐1α has been shown to decrease the expression of CD44 and Oct4, induce a reversal of the EMT phenotype, and suppress β‐catenin transcriptional activity in SW480 human CRC cells (Santoyo‐Ramos *et al.*, 2014). Moreover, HIF‐1α has been reported to play a promoting role in glycolysis which is highly activated in various CSCs than their differentiated counterparts (Emmink *et al.*, 2013). In good agreement, we found that *HIF‐1α* was not only a direct target of LRH‐1 (Fig. 3A) but its transcriptional activity was also markedly enhanced in certain population of the LRH‐1‐overexpressing clones (Fig. 3B) which could be accounted by the increases in both hypoxia and oxidative stress in these cells (Fig. 3C,D). Accordingly, the expression levels of some crucial glycolytic genes, the production of lactate, and the glycolytic capacity were rigorously elevated in the LRH‐1‐overexpressing clones (Fig. 4). Together, these results demonstrate that LRH‐1 stimulates glycolysis in human CRC cells mainly by activating HIF‐1α.

Since glycolysis was greatly increased in the LRH‐1‐overexpressing clones, we postulated that the mitochondrial function should be reduced in these cells. To our big surprise, the ROS levels (Fig. 5A), mitochondrial respiration as well as ATP production (Fig. 5B) were all markedly enhanced in the LRH‐1‐overexpressing clones. More strikingly, the expression levels of various subunits of different ETC complexes were also significantly elevated in them (Fig. 5C,D). To explain the increase of mitochondrial activity in the aforementioned clones, we next examined the effects of exogenous lactate because it has been shown to stimulate the expression of PGC‐1α, resulting in increased mitochondrial biogenesis and oxidative phosphorylation (OXPHOS) (Hashimoto *et al.*, 2007). As expected, marked increases in the expression levels of HIF‐1α, PGC‐1α, and MCT‐1 as well as the mitochondrial mass were detected in the wide‐type HCT‐116 and HT‐29 cells treated with exogenous lactate (Fig. 6A,B). In accordance, significantly higher protein levels of PGC‐1α and MCT‐1 as well as mitochondrial mass were also found in the LRH‐1‐overexpressing clones (Fig. 6C–E). To confirm the role of lactate in stimulating mitochondrial activity, we treated both the vector‐control and the LRH‐1‐overexpressing clones with 7ACC, a selective MCT‐1 inhibitor, and found that protein levels of the most aforementioned subunits of different ETC complexes were markedly decreased when the influx of lactate was blocked (Fig. 7A). Moreover, the ROS levels in the LRH‐1‐overexpressing but not the vector‐control clones were also drastically diminished by 7ACC treatment (Fig. S3). Subsequently, we demonstrated that the self‐renewal abilities of both the vector‐control and the LRH‐1‐overexpressing clones were dramatically reduced not only by 7ACC (Fig. 7B) but also by oxamate, an LDHA inhibitor, as well as rotenone, an ETC complex I inhibitor (Fig. S4A). Fittingly, the mRNA levels of CD133 and CD44 in the LRH‐1‐overexpressing clones were also decreased markedly by 7ACC treatment (Fig. 7C,D). As aforementioned, lactate (uptake) appears to be very important for the increased mitochondria biogenesis of some MCT‐1‐upregulating cells. However, a direct contribution of LRH‐1 in this process cannot be formally ruled out since a recent study has reported that the mitochondrial DNA copy number, basal respiration rate, ATP content, mitochondrial beta‐oxidation are significantly reduced in the hepatocytes isolated from the Lrh‐1 liver‐specific KO mice (Choi *et al.*, 2019). Unexpectedly, significant inhibition of the self‐renewal abilities of both the vector‐control and the LRH‐1‐overexpressing clones after being treated with ROS scavengers NAC (Fig. S4B) and ascorbic acid (Fig. S4C) were observed which indicates that the regenerative potential of CRCSCs is closely connected to intracellular ROS levels and cellular redox homeostasis. In good agreement, two earlier studies have already demonstrated that the regenerative potentials in both hematopoietic stem cells and neural stem cells were significantly abolished when intracellular ROS was suppressed below basal levels (Juntilla *et al.*, 2010; Le Belle *et al.*, 2011).

Finally, we found that OXPHOS but not glycolysis was the main metabolic pathway in the CD133^+^/CD44^+^ stem‐like subpopulations isolated from both the vector‐control and the LRH‐1‐overexpressing clones (Fig. 8), suggesting the coexistence of CRCSC subsets differing in their glucose utilization.

## 5 Conclusion

Our observations clearly demonstrate a ‘reverse Warburg Effect’ (Wilde *et al.*, 2017) in human CRCSCs because the expression levels and activity of HIF‐1α are markedly increased by LRH‐1 upregulation resulted from GATA6 overexpression in the majority of CRC cells which undergo ‘aerobic glycolysis’ (i.e., CD133^‐^/CD44^‐^, glycolytic, and stemness^lo^) to proliferate rapidly and, in the meantime, generate large amount of lactate that can be uptaken by another subset of CRC cells (i.e., MCT‐1^high^) for mitochondrial OXPHOS after being converted to pyruvate by LDHB (i.e., CD133^+^/CD44^+^, oxidative, and stemness^hi^) (Fig. S5). In this manner, metabolic symbiosis and energy transfer are maintained between two CRCSC subpopulations (e.g., CSC heterogeneity) which further supports the idea that simultaneous targeting glycolysis and mitochondrial metabolism with drug combinations might be the best approach to eradicate most, if not all, CRCSCs (Martinez‐Outschoorn *et al.*, 2017).

## Conflict of interest

The authors declare no conflict of interest.

## Author contributions

H‐TL and YS conceived and designed the study. H‐TL, C‐TC, and YS developed the methodology. H‐TL, W‐KT, C‐TC, T‐CC, and YS analyzed and interpreted the data (statistical analysis, biostatistics, computational analysis). H‐TL and YS wrote, reviewed, and/or revised the manuscript. H‐TL, W‐KT, and T‐CC made administrative, technical, or material support (reporting and organizing data, constructing databases). YS supervised the study.

## Supporting information


**Fig. S1.** The expression levels of four ESC markers are markedly increased in the LRH‐1 overexpressing clones. (A) The mRNA levels of four ESC markers (e.g., Oct4, Klf4, Nanog, and Sox2) in the vector‐control as well as the LRH‐1‐overexpressing HCT‐116 (left) and HT‐29 (right) clones were analyzed respectively by RT‐qPCR. (B) Total lysates (30 µg) prepared from various HCT‐116 (upper) and HT‐29 (lower) clones were subjected to immunoblot analysis using antibodies against the aforementioned four ESC markers as probes, respectively. GAPDH signals were used as loading controls. Data are mean ± S.D. from three independent experiments. *P < 0.05 compared with those of the corresponding vector‐control clones by Student's t‐test.

**Fig. S2.** Increased lactate levels in the LRH‐1 overexpressing clones are significantly diminished by oxamate treatment. (A) Cells lysates were prepared from the 116 Vec, OED, and OEJ clones (left) as well as the 29 Vec, OE7, and OE8 clones (right) after being seeded for 48 hrs and the intracellular lactate levels were measured as described. Data are mean ± S.D. from three independent experiments. *P < 0.05 compared with those of the corresponding vector‐control clones by Student's t‐test. The culture media (B) and total lysates (C) were collected from the aforementioned clones after they were treated without or with oxamate (5 mM) for 48 hr and the (B) extracellular as well as (C) intracellular lactate levels were respectively measured. Data (mean ± SD, N = 3) were analyzed by one‐way ANOVA with the LSD post hoc test and different characters represent different levels of significance (P < 0.05).

**Fig. S3.** The intracellular ROS levels in the LRH‐1‐overexpressing but not the vector‐control clones can be reduced significantly by 7ACC treatment. The intracellular ROS levels of the (A)116 Vec, OED, and OEJ clones as well as the (B) 29 Vec, OE7, and OE8 clones treated without or with 7ACC (20 nM) for 48 hr were determined by flow cytometry after they were stained with 1 mM DCFH‐DA. The gate population was measured by FlowJo V10 software.

**Fig. S4.** Suppression of glycolysis, oxidative phosphorylation, and ROS levels can decrease the self‐renewal abilities in the vector‐control as well as the LRH‐1‐overexpressing HCT‐116 and HT‐29 clones. Cells from three HCT‐116 and three HT‐29 clones were cultured respectively in defined media supplemented without or with 5 mM oxamate or 2 nM rotenone (A) and 1 mM NAC (B) as well as 1 or 5 mM ascorbic acid (AA) (C) for 20 days. Spheres stained by MTT were scanned and their numbers were counted by MetaMorph software. Data (mean ± SD, N = 3) were analyzed by one‐way ANOVA with the LSD post hoc test and different characters represent different levels of significance (P < 0.05).

**Fig. S5.** Proposed metabolic symbiosis between two CRCSC subpopulations.

**Table S1.** Nucleotide sequences of PCR primers.

## Data Availability

The data that support the findings of this study are available from the corresponding author upon reasonable request.
