# Supplementary figures and images for: GATA6 enhances the stemness of human colon cancer cells by creating a metabolic symbiosis through upregulating LRH‐1 expression
Source: Mol Oncol. 2020 Feb 26;14(6):1327–47. doi: 10.1002/1878-0261.12647 (PMC7266275; doi:10.1002/1878-0261.12647)

Supplemental Figure 1

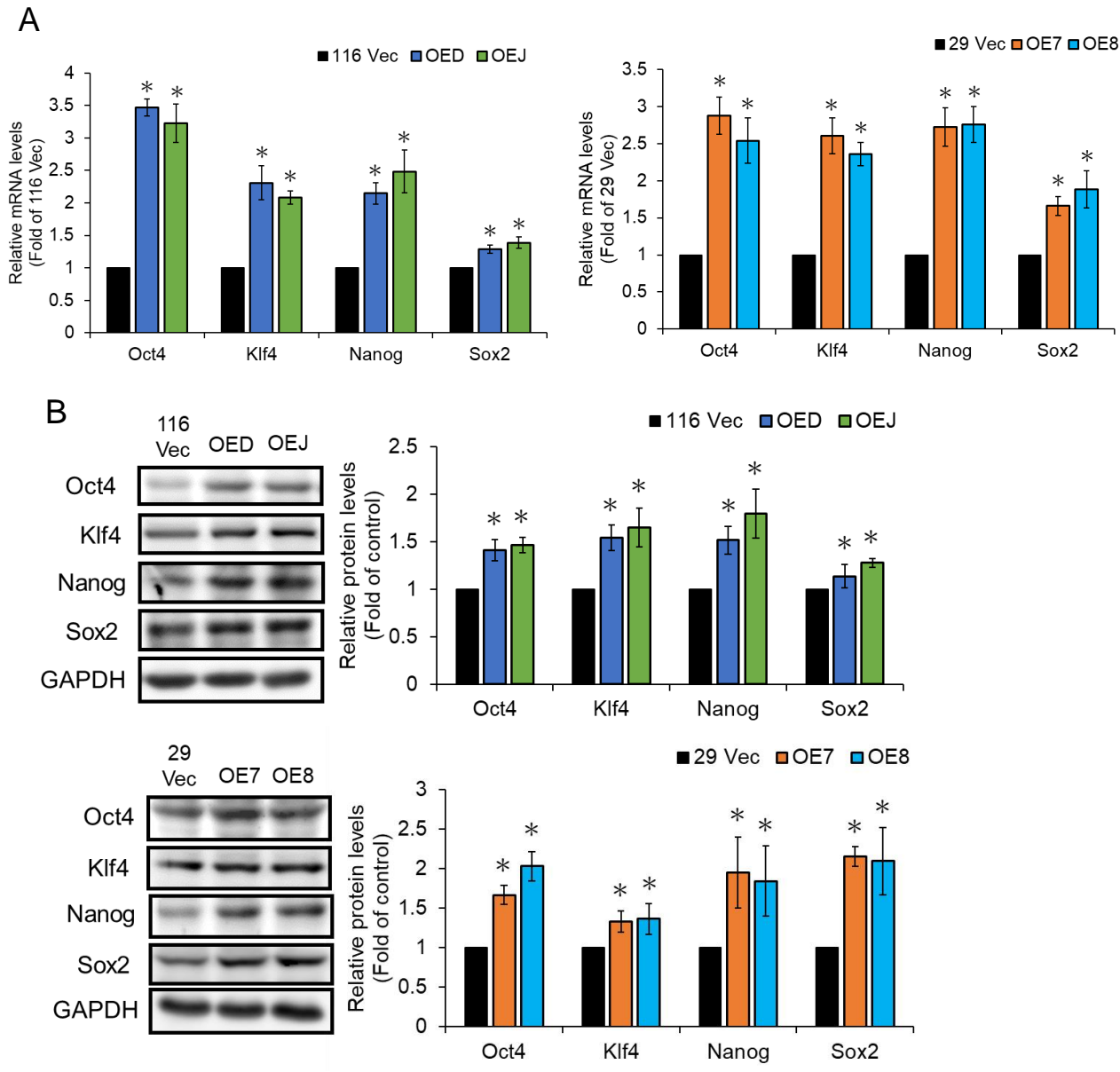

Supplement: Supplementary file 1 — Fig. S1. The expression levels of four ESC markers are markedly increased in the LRH‐1 overexpressing clones. (A) The mRNA levels of four ESC markers (e.g., Oct4, Klf4, Nanog, and Sox2) in the vector‐control as well as the LRH‐1‐overexpressing HCT‐116 (left) and HT‐29 (right) clones were analyzed respectively by RT‐qPCR. (B) Total lysates (30 µg) prepared from various HCT‐116 (upper) and HT‐29 (lower) clones were subjected to immunoblot analysis using antibodies against the aforementioned four ESC markers as probes, respectively. GAPDH signals were used as loading controls. Data are mean ± S.D. from three independent experiments. *P < 0.05 compared with those of the corresponding vector‐control clones by Student's t‐test. [file MOL2-14-1327-s001.pdf]

Supplemental Figure 2

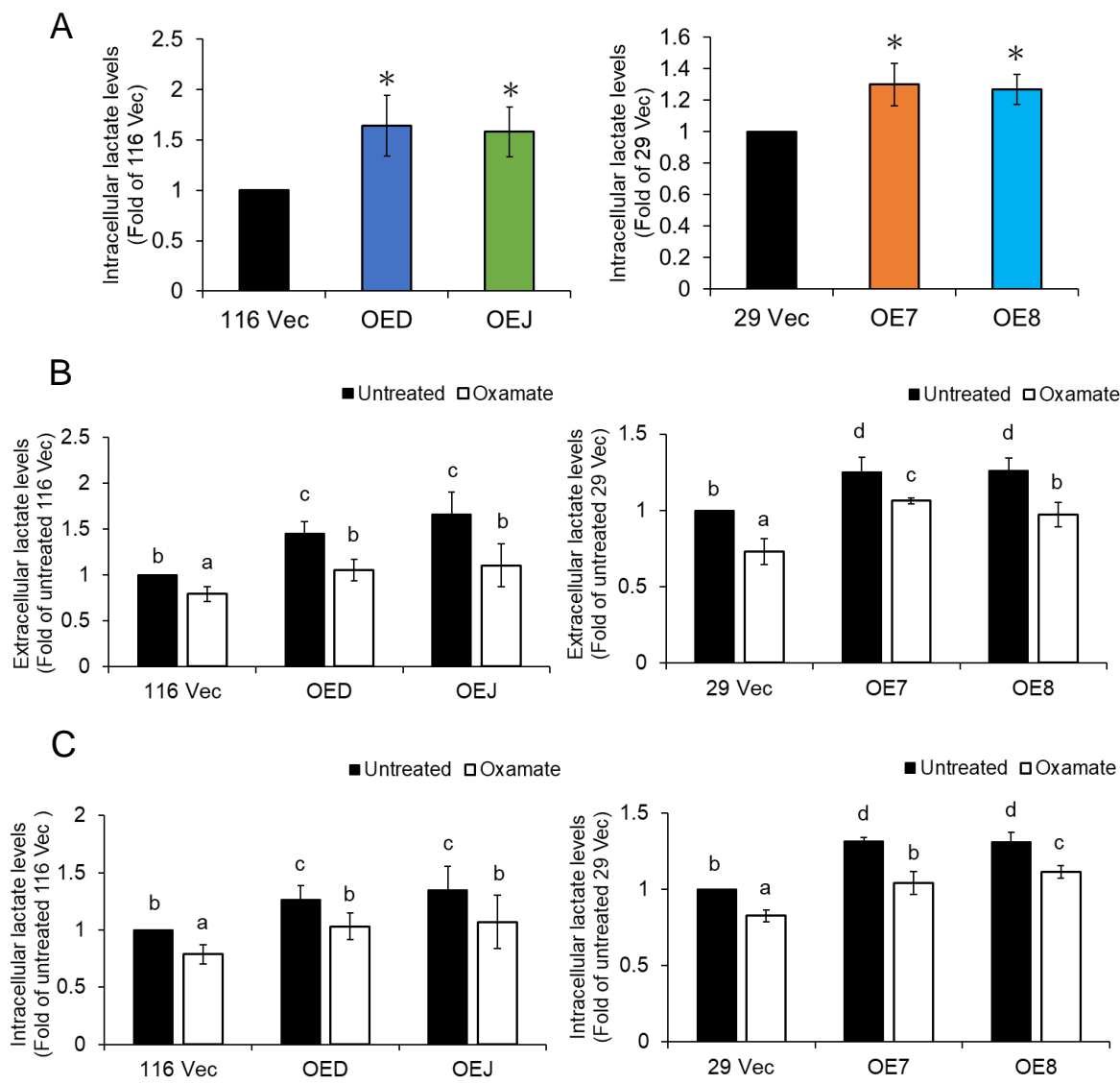

Supplement: Supplementary file 2 — Fig. S2. Increased lactate levels in the LRH‐1 overexpressing clones are significantly diminished by oxamate treatment. (A) Cells lysates were prepared from the 116 Vec, OED, and OEJ clones (left) as well as the 29 Vec, OE7, and OE8 clones (right) after being seeded for 48 hrs and the intracellular lactate levels were measured as described. Data are mean ± S.D. from three independent experiments. *P < 0.05 compared with those of the corresponding vector‐control clones by Student's t‐test. The culture media (B) and total lysates (C) were collected from the aforementioned clones after they were treated without or with oxamate (5 mM) for 48 hr and the (B) extracellular as well as (C) intracellular lactate levels were respectively measured. Data (mean ± SD, N = 3) were analyzed by one‐way ANOVA with the LSD post hoc test and different characters represent different levels of significance (P < 0.05). [file MOL2-14-1327-s002.pdf]

Supplemental Figure 3

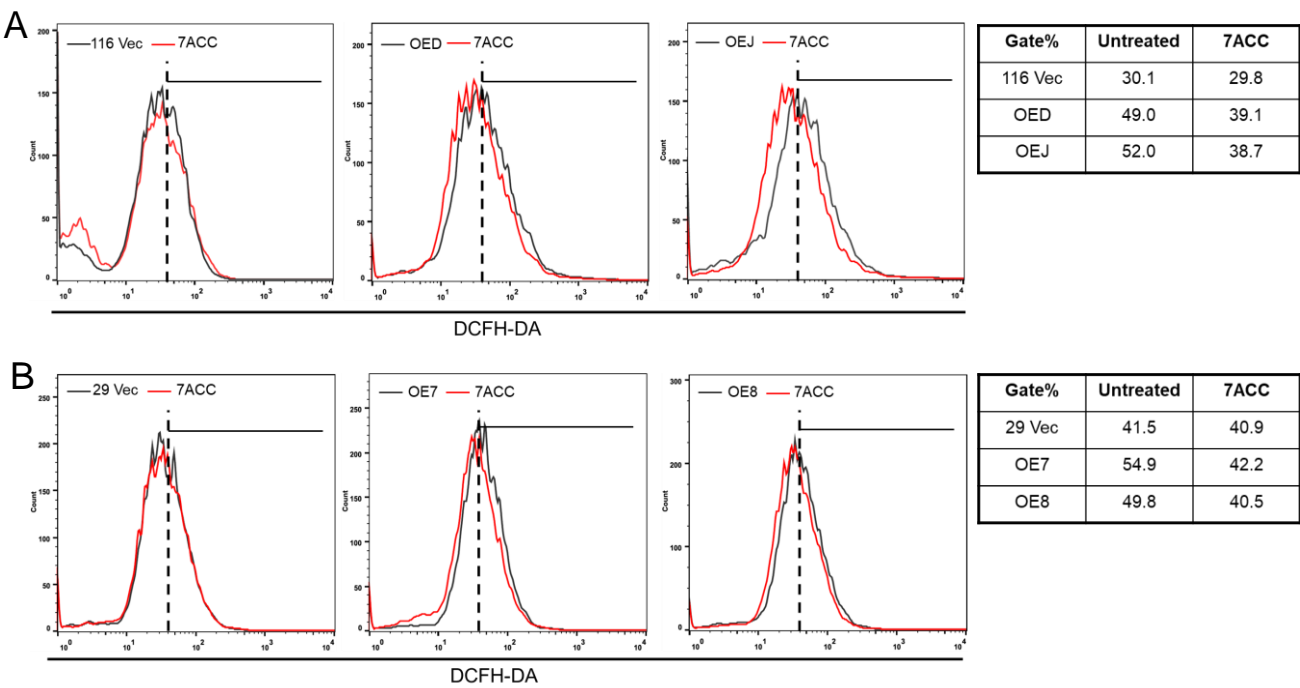

Supplement: Supplementary file 3 — Fig. S3. The intracellular ROS levels in the LRH‐1‐overexpressing but not the vector‐control clones can be reduced significantly by 7ACC treatment. The intracellular ROS levels of the (A)116 Vec, OED, and OEJ clones as well as the (B) 29 Vec, OE7, and OE8 clones treated without or with 7ACC (20 nM) for 48 hr were determined by flow cytometry after they were stained with 1 mM DCFH‐DA. The gate population was measured by FlowJo V10 software. [file MOL2-14-1327-s003.pdf]

Supplemental Figure 4

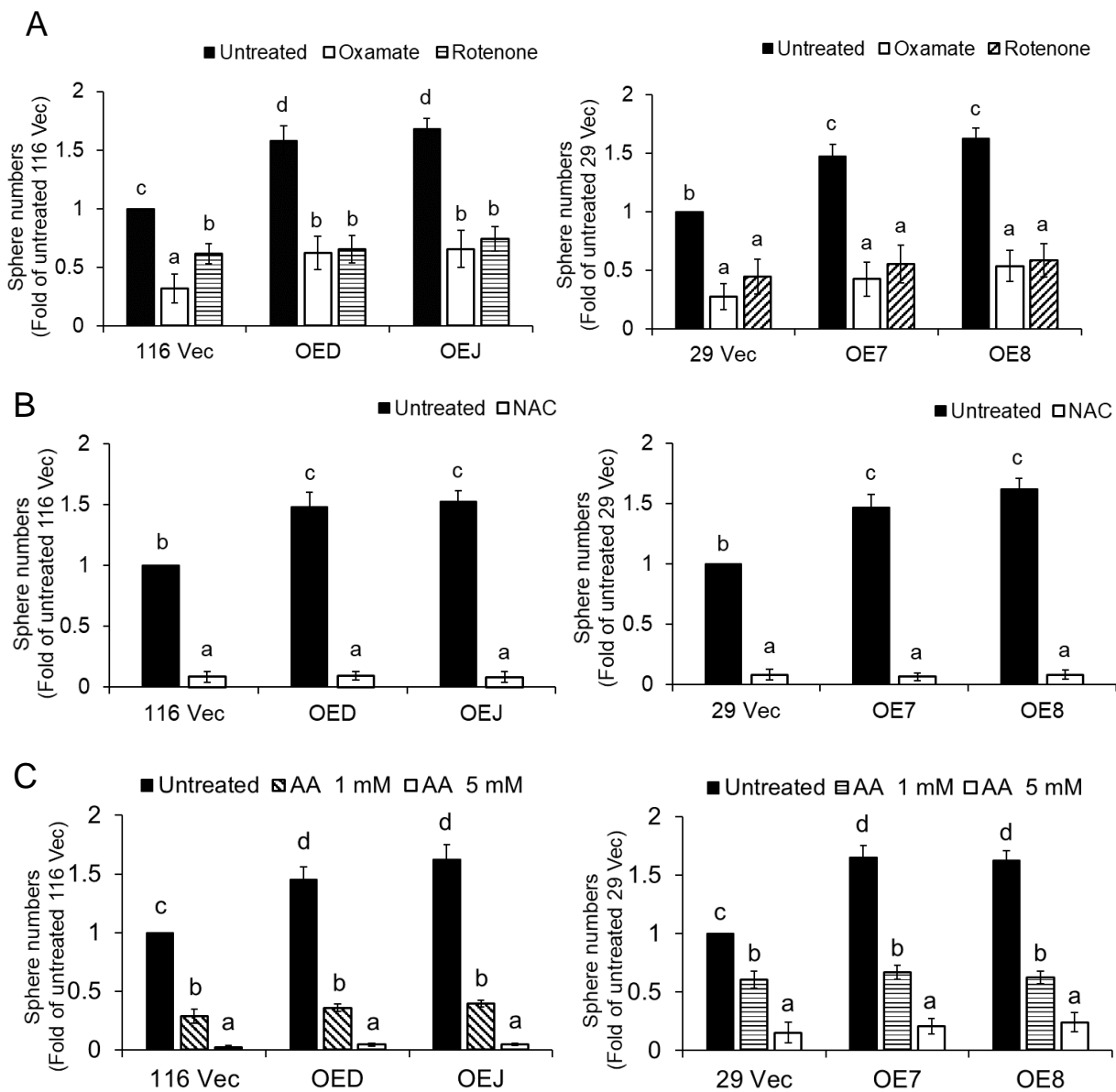

Supplement: Supplementary file 4 — Fig. S4. Suppression of glycolysis, oxidative phosphorylation, and ROS levels can decrease the self‐renewal abilities in the vector‐control as well as the LRH‐1‐overexpressing HCT‐116 and HT‐29 clones. Cells from three HCT‐116 and three HT‐29 clones were cultured respectively in defined media supplemented without or with 5 mM oxamate or 2 nM rotenone (A) and 1 mM NAC (B) as well as 1 or 5 mM ascorbic acid (AA) (C) for 20 days. Spheres stained by MTT were scanned and their numbers were counted by MetaMorph software. Data (mean ± SD, N = 3) were analyzed by one‐way ANOVA with the LSD post hoc test and different characters represent different levels of significance (P < 0.05). [file MOL2-14-1327-s004.pdf]

Supplemental Figure 5

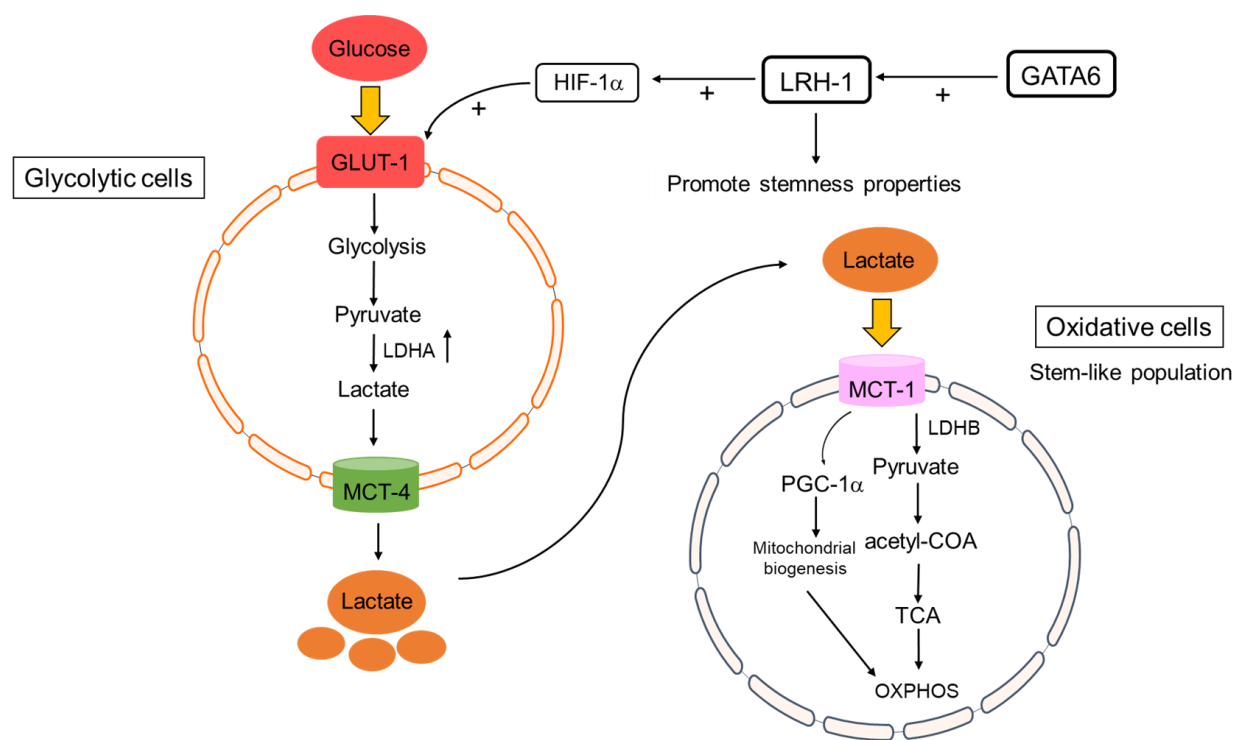

Supplement: Supplementary file 5 — Fig. S5. Proposed metabolic symbiosis between two CRCSC subpopulations. [file MOL2-14-1327-s005.pdf]
