# Supplementary material for: GATA6 enhances the stemness of human colon cancer cells by creating a metabolic symbiosis through upregulating LRH‐1 expression
Source: Mol Oncol. 2020 Feb 26;14(6):1327–47. doi: 10.1002/1878-0261.12647 (PMC7266275; doi:10.1002/1878-0261.12647)
Supplement: Supplementary file 6 — Table S1. Nucleotide sequences of PCR primers. [file MOL2-14-1327-s006.pdf]

Supplemental Table 1. Nucleotide sequences of PCR primers

Gene	Sequence
ChIP-LRH-1	F: 5'CTGTGCTGCATAGCACCTCT-3'<br>R: 5'GCCTTGGGAAGGACACATCA-3'
LRH-1	F: 5'CTGATACTGGAACTTTTGAA-3'<br>R: 5'TACTACTCCTGGCCCACTCG-3'
CD44	F: 5'-TGGCACCCGCTATGTCCAG-3'<br>R: 5'-GTAGCAGGGATTCTGTCTG-3'
CD133	F: 5'-TTCTTGACCGACTGAGACCCA-3'<br>R: 5'-TCATGTTCTCCAACGCCTCTT-3'
LGR5	F: 5'-TGCTGGCTGGTGTGGATGCG-3'<br>R: 5'-GCCAGCAGGGCACAGAGCAA-3'
ALDH-1	F: 5'-GCACGCCAGACTTACCTGTC-3'<br>R: 5'-CCTCCTCAGTTGCAGGATTAAAG-3'
Ascl2	F: 5'-GTGAAGCTGGTGAACTTGGGC-3'<br>R: 5'-CAGCGTCTCCACCTTGCTCA-3'
OCT4	F: 5'-GAGAACCGAGTGAGAGGCAACC-3'<br>R: 5'-CATAGTCGCTGCTTGATCGCTTG-3'
KLF4	F: 5'-ACCAGGCACTACCGTAAACACA-3'<br>R: 5'-GGTCCGACCTGGAAAATGCT-3'
Nanog	F: 5'-AATACCTCAGCCTCCAGCAGATA-3'<br>R: 5'-TGCGTCACACCATTGCTATTCTTC-3'
SOX2	F: 5'-CCCTGCTGAGAATAGGACAT-3'<br>R: 5'-CCCTGCAGTACAACTCTATG-3'
HIF-1α	F: 5'-TGATGACCAGCAACTTGAGG-3'<br>R: 5'-CTGGGGGCATGGTAAAAGAAA-3'
GLUT-1	F: 5'- CCTGCAGTTTGGCTACAACA-3'<br>R: 5'- TAACGAAAAGGCCCACAGAG-3'
LDHA	F: 5'-GAGGTTCACAAGCAGGTGGT-3'<br>R: 5'-CCCAAAATGCAAGGAACACT-3'
PDK-1	F: 5'-GCTCTCCATGAAGCAGTTCC-3'<br>R: 5'-ACCAATTGAACGGATGGTGT-3'
MCT-1	F: 5'-TCCAGCTCTGACCATGATTG-3'<br>R: 5'-GCCCCCAAGAATTAGAAAGC-3'
MCT-4	F: 5'-GCACCCACAAGTTCTCCAGT-3'<br>R: 5'-CAAAATCAGGGAGGAGGTGA-3'
NDUFB8	F: 5'-CCCTATCCTAGGACCCCAGA-3'

	R: 5’-TCCACACGGTTCCTGTTGTA-3’
SDHB	F: 5’-GGAAGGCAAGCAGCAGTATC-3’<br>R: 5’-AGCGATAGGCCTGCATAAGA-3’
UQCRC2	F: 5’-ATGGCTTTGATTGGACTTGG-3’<br>R: 5’-CAAAAGCAGCATGGACAAGA-3’
MTCO1	F: 5’-ACGTTGTAGCCCACTTCCAC-3’<br>R: 5’-CATCGGGGTAGTCCGAGTAA-3’
ATP5A	F: 5’-CATTGTGGACGTTCCAGTTG-3’<br>R: 5’-ATTGGCACCAAGCTATCCAC-3’
PGC-1α	F: 5’-GTGAAGACCAGCCTCTTTGC-3’<br>R: 5’-AATCCGTCTTCATCCACAGG-3’
β-actin	F: 5’-TGGCATTGCCGACAGGAT-3’<br>R: 5’-GCTCAGGAGGAGCAATGATCT-3’
